# Robust fuzzy sliding mode controller for a skid-steered vehicle subjected to friction variations

**DOI:** 10.1371/journal.pone.0258909

**Published:** 2021-11-16

**Authors:** Yasir Mehmood, Jawad Aslam, Nasim Ullah, Ahmed A. Alsheikhy, Emad Ud Din, Jamshed Iqbal

**Affiliations:** 1 School of Mechanical and Manufacturing Engineering, National University of Sciences and Technology, Islamabad, Pakistan; 2 Department of Electrical Engineering, College of Engineering, Taif University, Taif, Kingdom of Saudi Arabia; 3 Department of Electrical Engineering, College of Engineering, Northern Border University, Arar, Saudi Arabia; 4 Department of Computer Science and Technology, Faculty of Science and Engineering, University of Hull, Hull, United Kingdom; J.C. Bose University of Science and Technology, YMCA, INDIA

## Abstract

Skid-steered vehicles (SSV) are gaining huge importance in the market due to their applications like construction, agricultural work, material handling etc. The accuracy of performing such tasks require a robust control algorithm. The design of such controller is very challenging task due to external disturbances caused by wheel-ground interaction and aerodynamic effects. This paper proposes robust fractional and integral order fuzzy sliding mode controllers (FSMC, FFSMC) for a skid-steered vehicles with varying coefficient of friction and a displaced center of gravity (CG). FFSMC controller reduces the outcome of forces generated as a result of ground tire interaction during skidding and friction variations. The proposed controllers are implemented for a four-wheel SSV under high-speed turning motion. A simulation environment is constructed by implementing the SSV dynamics with wheel-road model and the performance of the proposed algorithms is tested. The simulation test is conducted for a Pioneer-3AT (P-3AT) robot SSV vehicle with displaced CG and variable coefficient of tires friction. Simulation results demonstrate the efficiency of the proposed FFSMC algorithm in term of reduced state errors and minimum chattering. The proposed controller compensates the effect of different responses of the wheels generated as a result of variable CG. The chattering phenomenon generated by conventional SMCs is also minimized by fuzzy tuning approach.

## Introduction

Motion control of skid-steered vehicle (SSV) is a challenging task due to the nonlinearities arising as a result of slip and braking phenomena. The undetected and immediate coefficient of friction introduces uncertainties in the vehicle dynamics. The change in coefficient of friction due to the change in surface greatly affects the wheel dynamics. It also causes unequal angular accelerations at SSV tires which results in vehicle skidding. Thus, in the absence of an optimal controller design, automatic guidance of SSV is a challenging task. In the existing literature, the modeling and control design of Ackermann-steered and differential drive vehicle have been extensively reported but less work has been done on four-wheeled differential drive vehicles.

Wheel-road interaction is an important factor as it influences the desired vehicle dynamics such as wheel-ground forces and torques [1]. The projections of forces between wheel and road at a contact point is modeled in the form of components [2]. The braking and steering forces are the sub-components of the force, which is highly dependent on the wheel-road contact plane. Similarly, normal forces are distributed in the contact plane and are perpendicular to the road. Slip angle and wheel slip are used to measure wheel forces and torques [3]. SSV model is derived by integrating the tire model into the vehicle dynamics model [4]. Semi-empirical tire model requires a smaller number of inputs as compared to other models and they are computationally efficient, one example of which is TM-easy model [5]. With the change in coefficient of friction, the maximum tire force and the full sliding force change but the initial inclination remains unaffected. The TM-easy tire parameters vary by changing the tire-road combination [6]. When the location of CG does not coincide with the geometric center of SSV, it effects the parameters of the SSV model [7].

In the reported literature, several methods have been proposed to control the longitudinal velocity and yaw angle of the SSV. A skid-steered vehicle is controlled through vision-based target tracking technique using guided policy search which is based on the general kinematics slip model and a field of view constraint in [8]. A robust controller is developed for an SSV to ensure high speed path following in [9]. A non-linear control law guarantees the convergence of SSV to the path while a kinematic model describes the terrain dependent motion of SSV. A model predictive controller is proposed for the path tracking of SSV with an online model learning in [10]. Here, the velocity model is learned with an online sparse Gaussian process. A Sampling Based Model Predictive Optimization algorithm is proposed for SSV in [11] to plan paths which are energy efficient for mixed surface types operational areas. A major drawback of SMC method is the high frequency chattering phenomenon due to its discontinuous control part [12]. Lucet et al. [13, 14] proposed a robust algorithm for the compensation of skidding phenomena in SSV. However, in the aforementioned methods, the increase in power consumption due to chattering phenomena was reported. In order to minimize chattering, a boundary layer design was introduced in [15], but the suggested method degrades steady state error. A fuzzy logic control (FLC) is proposed for accurate tracking of longitudinal velocity and yaw angle of SSV in [16, 17]. A model-based coordinated adaptive robust tracking controller is proposed in [18], which generates the motor driving torque commands for the four wheels of SSV and consists of three-level control architecture. An online estimation of the location of track instantaneous centers of rotation of SSV and its modeled based motion prediction is achieved by a kinematic extended Kalman filter in [19]. The combination of FLC and SMC generates a sliding mode FLC controller that exploits the benefits of adaptive tuning and robustness. A fuzzy SMC (FSMC) is investigated in [20] to improve transient response of a nonlinear system. The FSMC is advantageous because tracking error is minimized and chattering phenomenon of traditional SMC is also reduced. To improve the stability of SSV vehicle, a hybrid FSMC algorithm is applied in [21, 22]. Numerical optimal control methods for backlash compensation of electric powertrains of electric vehicles have been studied in [23]. In [24], a novel method is presented to estimate the nonlinear backlash phenomena. Similarly in [25], a Hardware in Loop (HIL) test bench is reported for conventional braking system with pressure following strategy.

The above literature mostly reports integer order control methods for the SSV vehicles. In recent times, fractional calculus is widely applied in different control problems. The most widely reported schemes include fractional order classical proportional integral derivative (FOPID) controllers [26]. Fractional order schemes offer more degree of freedom to adjust system response as compared to the integer order controllers. A fractional order PID controller is discussed in [26] for the trajectory tracking of a ground vehicle and its effectiveness is verified using simulations tests. Similarly, the experimental verification of the effectiveness of a fractional order PI controller for a four-wheeled SSV is discussed in [27]. A fractional order PID controller is reported for generating the standard inputs in a four-wheeled differential drive applications [28]. Apart from the usual applications of fractional order calculus in control formulations, it is very important to highlight some of its benefits such as robustness to noise and disturbances, wide stability margins and its inherent memory. In [29], a detailed survey is given for highlighting the benefits of the fractional order controllers. Similarly, in [30, 31], robust fractional order PID controllers are developed for a nonlinear uncertain system.

Based on the cited literature, in this paper, as a first step, integer order SMC controller is formulated based on the longitudinal and yaw dynamics of a SSV. Later on, the idea is extended to formulate a fractional order SMC controller. Finally, the discontinuous control parts of the integral and fractional order SMC methods are approximated using fuzzy logic system. The proposed controllers are investigated for SSV vehicle subjected to variations in friction coefficient and CG. The control algorithms are tested in the simulation environment on different ground surfaces and with displaced CG. Specific contributions of this work are highlighted as follows:

1. Fractional order robust control schemes are rarely exploited for the SSV vehicles. In this paper, a fuzzy gain supervisor based fractional order SMC controller is derived and tested for the longitudinal velocity and yaw angle control of a SSV.
2. In our proposed design, the discontinuous term contains a fractional integrator. i.e. *D*^−*α*^ *sgn*(.), so fractional integrator is adjusted to smooth out the oscillations in the control torque and tracking signals, while preserving the robustness of the controller.
3. The fractional order control is dependent on the fractional order derivative of the yaw angle instead of yaw rate. So using the idea presented in [29], a fractional operator is robust to the measurement noise and thus, yaw rate is no more required.

## Mathematical modeling of a four-wheel skid-steered vehicle

### Tire model of SSV

**Fig 1:**
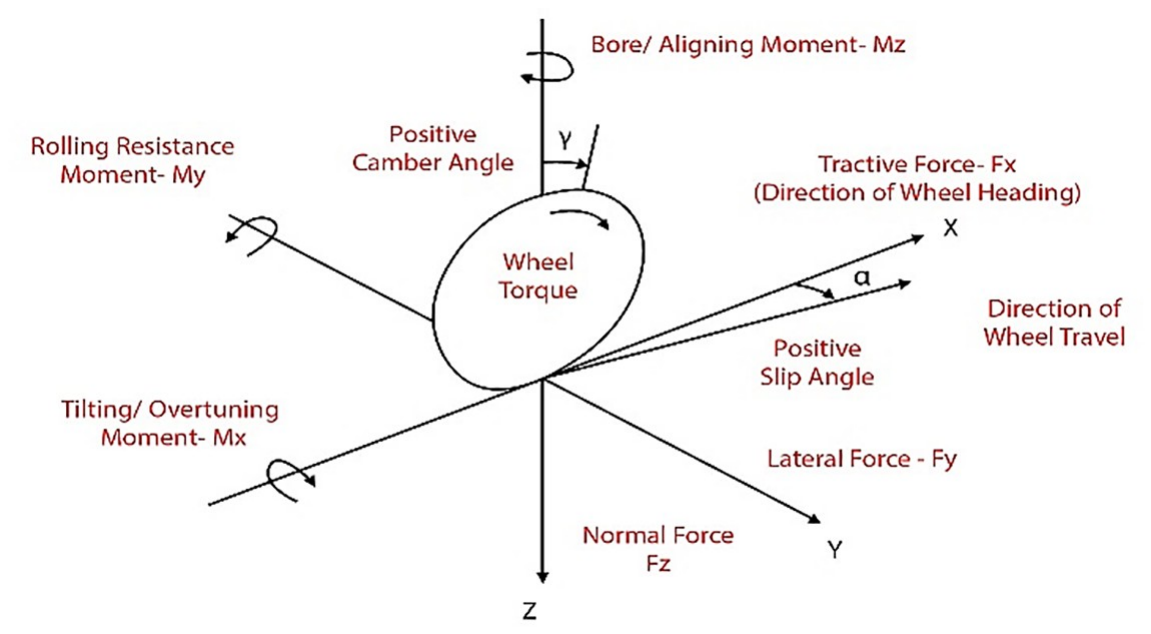
SAE tire axis coordinate system.

In dynamic modeling of SSV, wheel modelling is very important as it defines the forces produced during tire road interaction. Along with gravitational and aerodynamic forces, the forces and moments generated as a result of ground tire interaction normally control the motion of the SSV. Hence, these forces are to be calculated to derive the vehicle dynamics. Numerous methods have been utilized for prediction of forces and moments from the available data on road tire interaction. A reference tire axis system has been recommended by Society of Automotive Engineers (SAE) as shown in Fig 1. The figure shows all the forces and moments at a contact point, where *x* is referred as longitudinal direction, *y* as lateral direction and *z* direction is normal to the plane.

**Fig 2:**
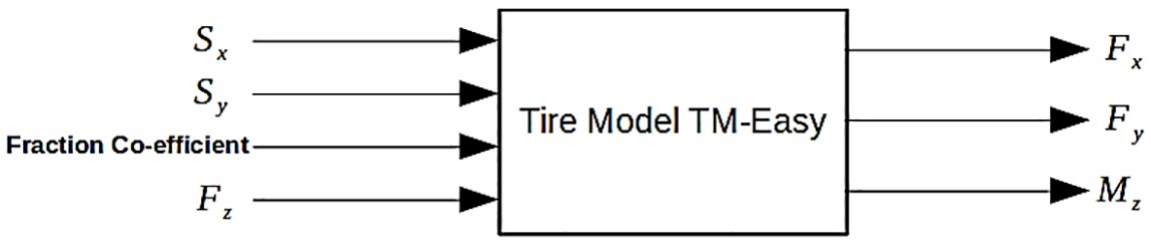
Inputs and outputs of TM-easy tire model [7].

This paper examines the tire model, known as TM-easy tire model, for simulations of a SSV system. Negligible contact moments and zero camber are considered. The tire is considered as a rigid disc; hence the radius of the tire remains constant. The forces in longitudinal direction are modelled and measured as a function of slip variable, while the lateral direction forces are modelled as a function of slip angle. The aligning moment is defined as a product of pneumatic trail and lateral force. The inputs and outputs of TM-easy tire model are shown in Fig 2 [7].

**Fig 3:**
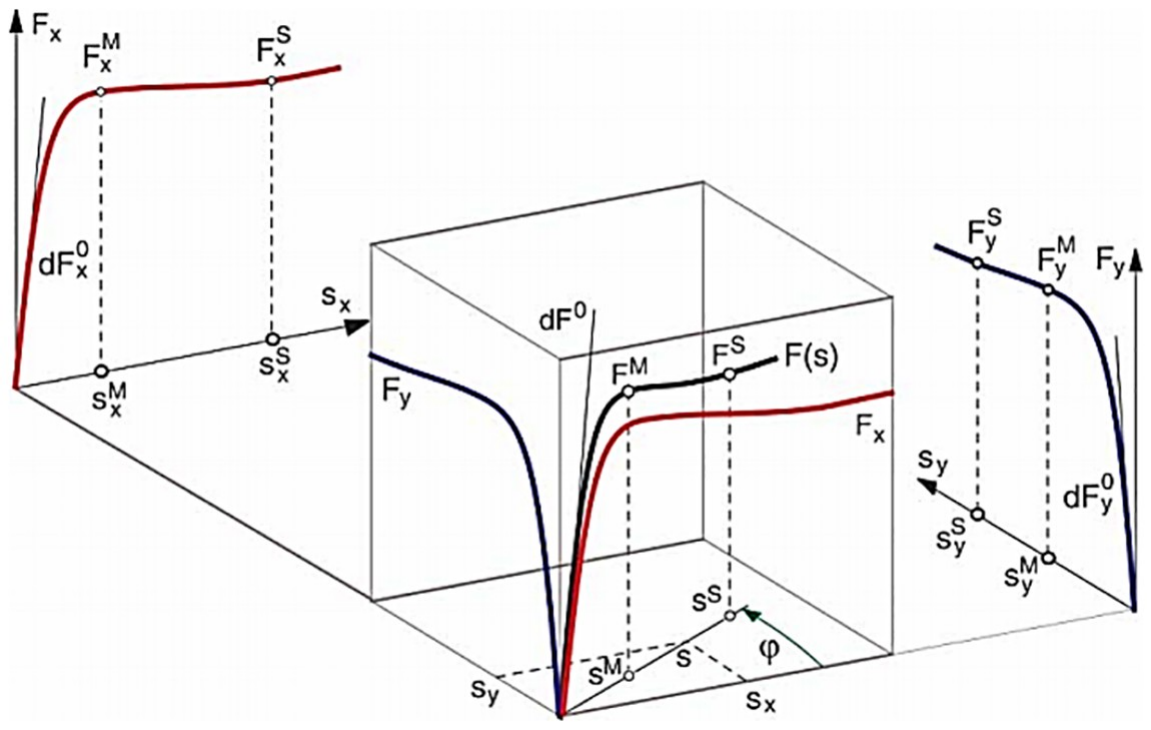
Generalized tire parameters [6].

The longitudinal and lateral slips of TM-easy tire model are respectively defined in (1) and (2):

$$\begin{array}{c} S_{x}=\;\frac{Vx-R\omega }{R\omega } \end{array}$$ (1)

$$\begin{array}{c} S_{y}=\frac{Vy}{R\omega } \end{array}$$ (2)

where the longitudinal and lateral velocities are *V*_*x*_ and *V*_*y*_, and the angular velocity is *ω*. Slip varies from 0 to ∞. The tire characteristic parameters *dF*^0^, *S*^*M*^, *F*^*M*^, *S*^*f*^, *F*^*f*^ define the tire forces and are shown in Fig 3 [6]. The longitudinal and lateral force characteristics are used for the calculation of these parameters. *F*_*x*_ and *F*_*y*_ act as functions of *S*_*x*_ and *S*_*y*_ respectively, and are defined by their corresponding characteristic parameters. The parameters $dF_{x}^{0}$ and $dF_{y}^{0}$ represent the initial slopes, $S_{x}^{M}$ and $S_{y}^{M}$ are the slips at maximum forces $F_{x}^{M}$ and $F_{y}^{M}$. $S_{x}^{f}$ and $S_{y}^{f}$ represent the sliding limits at full sliding force $F_{x}^{f}$ and $F_{y}^{f}$.

**Fig 4:**
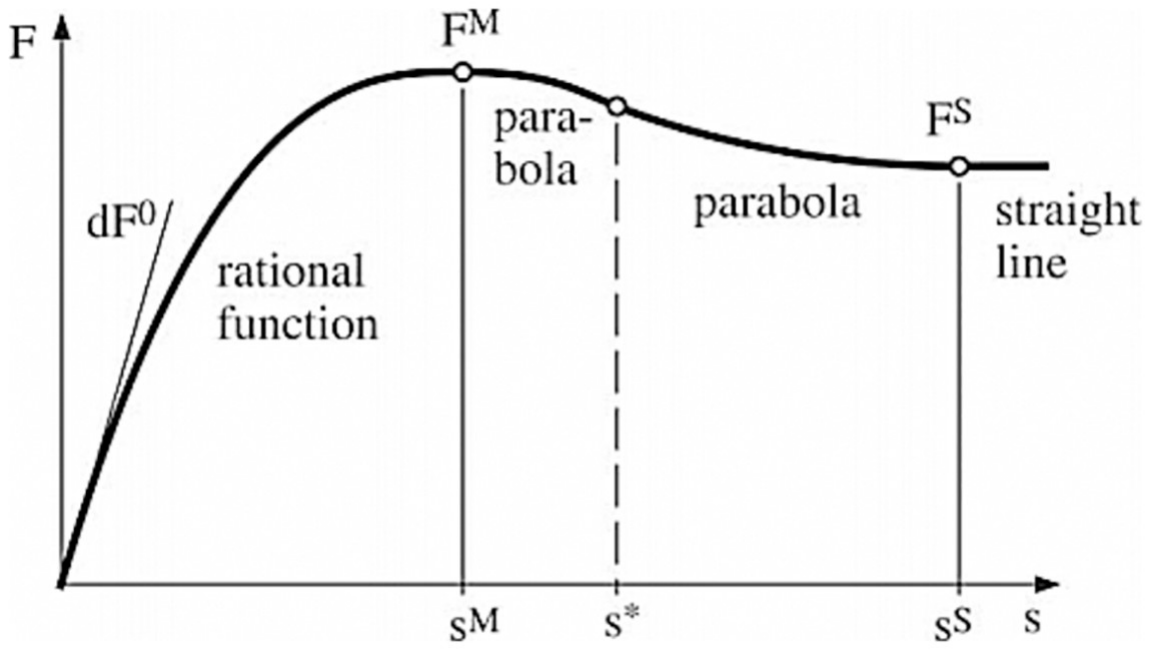
Response of tire force to slip [6].

The generalized tire force is calculated in the intervals defined in Fig 4 [6] by an appropriate function. A rational function, which is defined by the inclination, maximum slip and tire force is used in the first interval. Then, the tire force parameters are varied in a parabolic shape until the full sliding area is reached, after which the curve continues in a straight line.

In order to determine the combined braking and steering forces, normalized and slightly modified longitudinal and lateral slips are calculated, which are added to further compute the combined slip variable as given below [7].

$$\begin{array}{c} S_{xy\;}=\;\sqrt{{\left(\frac{S_{x}}{{\hat{S}}_{x}}\right)}^{2}+{\left(\frac{S_{y}}{{\hat{S}}_{y}}\right)}^{2}} \end{array}$$ (3)

$$\begin{array}{c} {\hat{S}}_{x}=\;\frac{S_{x}^{M}}{S_{x}^{M}+S_{y}^{M}}+\frac{F_{x}^{M}{dF}_{x}^{0}}{F_{x}^{M}{dF}_{x}^{0}+F_{y}^{M}{dF}_{y}^{0}} \end{array}$$ (4)

$$\begin{array}{c} {\hat{S}}_{y}=\;\frac{S_{y}^{M}}{S_{x}^{M}+S_{y}^{M}}+\frac{F_{y}^{M}{dF}_{y}^{0}}{F_{x}^{M}{dF}_{x}^{0}+F_{y}^{M}{dF}_{y}^{0}} \end{array}$$ (5)

where ${\hat{S}}_{x}$ and ${\hat{S}}_{y}$ are normalized longitudinal and lateral slips respectively. The combined tire force is defined by model parameters, which depend on longitudinal and lateral forces. Now, the generalized tire force for all the three intervals of slips variable is calculated by (6)–(8) as follows.

$$\begin{array}{cc} F\;(S_{x},\;S_{y})=\frac{\sigma S^{M}{dF}^{0}}{1+\sigma (\sigma +\frac{S^{M}}{F^{M}}F^{f}-2)}\;\;\;\;\; & for\;\;\;\;\;0<S_{xy}<S^{M} \end{array}$$ (6)

Here, $\sigma =\frac{S_{xy}}{S^{M}}$

$$\begin{array}{cc} F(S_{x},S_{y})=F^{M}-(F^{M}-F^{f})\sigma^{2}(3-2\sigma )\;\;\;\;\;\; & for\;\;\;\;\;S^{M}<S_{xy}<S^{f} \end{array}$$ (7)

Here, $\sigma =\frac{S_{xy}-S^{M}}{S^{f}-S^{M}}$

$$\begin{array}{cc} F(S_{x},S_{y})=F^{f}\;\;\;\;\; & for\;\;\;\;\;S_{xy}\geq S^{f} \end{array}$$ (8)

All other parameters used in the combined force calculations of TM-easy tire model are defined in (9)–(13).

$$\begin{array}{c} {dF}^{0}=\sqrt{{\left({dF}_{x}^{0}\hat{S_{x}}cos\varphi \right)}^{2}+{\left({dF}_{y}^{0}\hat{S_{y}}sin\varphi \right)}^{2}} \end{array}$$ (9)

$$\begin{array}{c} S^{M}=\sqrt{{\left(\frac{S_{x}^{M}}{\hat{S_{M}}}cos\varphi \right)}^{2}+{\left(\frac{S_{y}^{M}}{\hat{S_{M}}}sin\varphi \right)}^{2}} \end{array}$$ (10)

$$\begin{array}{c} F^{M}=\sqrt{{\left(F_{x}^{M}cos\varphi \right)}^{2}+{\left(F_{y}^{M}sin\varphi \right)}^{2}} \end{array}$$ (11)

$$\begin{array}{c} S^{f}=\sqrt{{\left(\frac{S_{x}^{f}}{\hat{S_{x}}}cos\varphi \right)}^{2}+{\left(\frac{S_{y}^{f}}{\hat{S_{y}}}sin\varphi \right)}^{2}} \end{array}$$ (12)

$$\begin{array}{c} F^{f}=\sqrt{{F_{x}^{f}(cos\varphi )}^{2}+{\left(F_{y}^{f}sin\varphi \right)}^{2}} \end{array}$$ (13)

Finally, the components of longitudinal and lateral forces are derived from the projections in longitudinal and lateral directions i.e.

$$\begin{array}{c} F_{x}(S_{x},S_{y})=F(S_{x},S_{y})cos\varphi \end{array}$$ (14)

$$\begin{array}{c} F_{y}(S_{x},S_{y})=F(S_{x},S_{y})sin\varphi \end{array}$$ (15)

$$\begin{array}{cc} cos\varphi =\frac{\frac{S_{x}}{\hat{S_{x}}}}{S_{xy}};\;\;\;\;\;\;\;\; & sin\varphi =\frac{\frac{S_{y}}{\hat{S_{y}}}}{S_{xy}} \end{array}$$ (16)

Eqs (14)–(16) are simplified as (17) and (18).

$$\begin{array}{c} F_{x}=F\frac{\frac{S_{x}}{\hat{S_{x}}}}{S}=\frac{F}{S}\left(\frac{S_{x}}{\hat{S_{x}}}\right)=f\left(\frac{S_{x}}{\hat{S_{x}}}\right) \end{array}$$ (17)

$$\begin{array}{c} F_{y}=F\frac{\frac{S_{y}}{\hat{S_{y}}}}{S}=\frac{F}{S}\left(\frac{S_{y}}{\hat{S_{y}}}\right)=f\left(\frac{S_{y}}{\hat{S_{y}}}\right) \end{array}$$ (18)

where *f* represents the global derivative of F.

### Influence of friction coefficient on tire parameters

The tire parameters are only true for one specific tire-road combination. Hence, by changing the tire-road combination, the tire model parameters also change. Tire-road combination also affects the coefficient of friction. Changing coefficient of friction mainly affects the maximum tire force and the full sliding force while the inclination remains unaffected. Hence;

$$\begin{array}{c} S_{new}^{M}=\frac{\mu_{new}}{\mu_{old}}S_{old}^{M},\;\;\;\;\;\;\;F_{new}^{M}=\frac{\mu_{new}}{\mu_{old}}F_{old}^{M},\;\;\;\;\;\;\;S_{new}^{f}=\frac{\mu_{new}}{\mu_{old}}S_{old}^{f},\;\;\;\;\;\;\;F_{new}^{f}=\frac{\mu_{new}}{\mu_{old}}F_{old}^{f} \end{array}$$

This means that tire model parameters depend on friction coefficient and the new coefficient.

### SSV dynamics model

**Fig 5:**
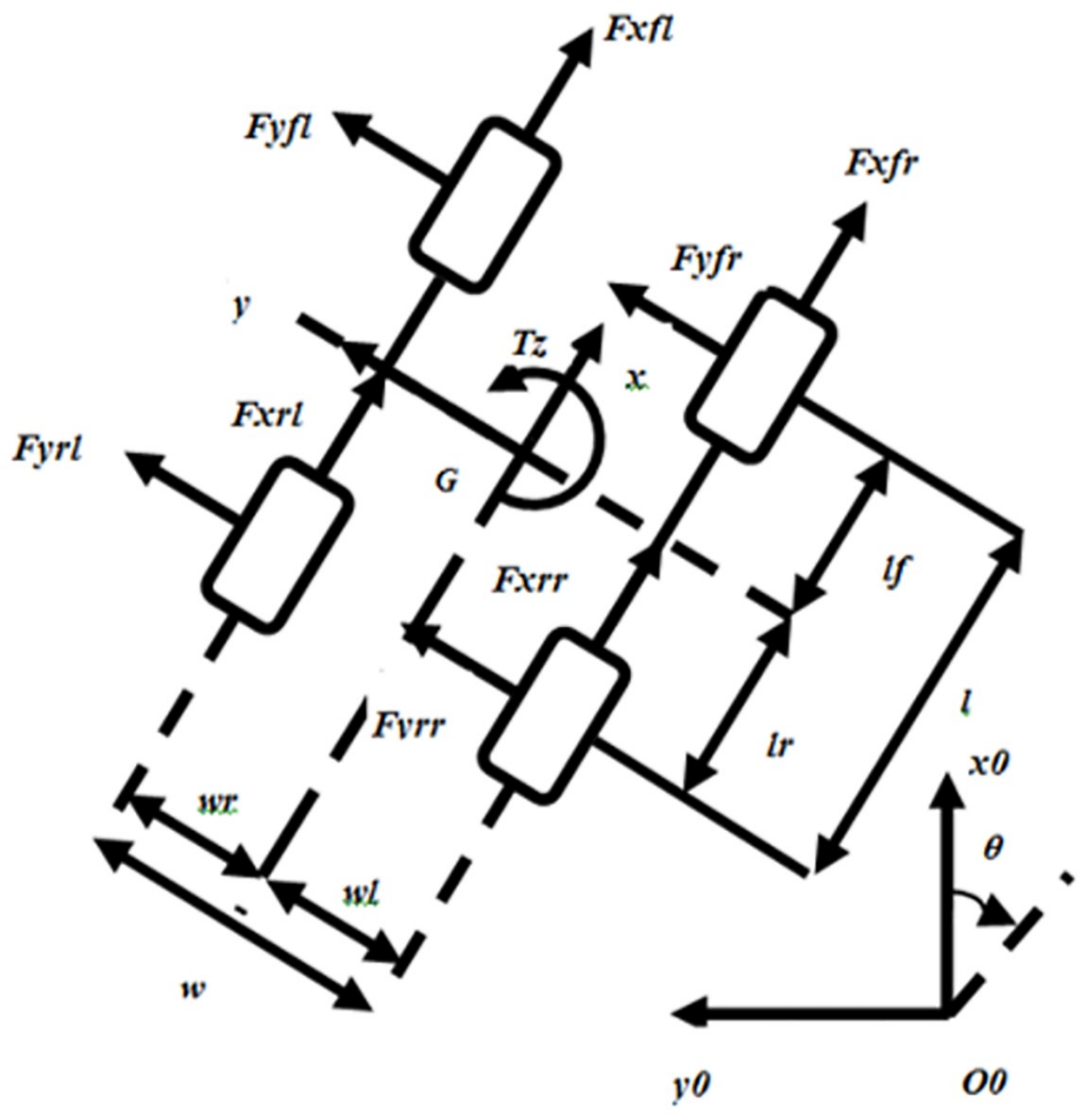
Four-wheel SSV dynamic model [6].

The dynamic modelling of SSV is required for the model-based controller design. The dynamic model of the SSV vehicle is derived in global frame of reference *R*_0_ = [*O*_0_, *x*_0_, *y*_0_, *z*_0_]. Local frame of reference is represented by *R* = [*G*, *x*, *y*, *z*_0_] and [*x*, *y*, *θ*]^*T*^ represents the position vector of SSV. Position of CG is given at [*x*, *y*]^*T*^. The dynamics of four-wheel SSV is shown in Fig 5. Before formulating the dynamic model of SSV, the following assumptions are made.

**Assumption 1**: The vehicle is assumed to be moving at a slow speed in an indoor environment, so the aerodynamic effects are neglected. The maximum speed assumed here is 2 m/sec.
**Assumption 2**: The tire is assumed to be rigid; hence the rolling resistance is neglected as it is caused by deformation of tire or surface.
**Assumption 3**: The tire is considered as a rigid disc; hence the radius of the tire remains constant.
**Assumption 4**: Due to the small size, low weight and stiffness of the suspension system, roll and pitch motion of the robotic vehicle are neglected [32].

The mapping from local to global frame of reference is given by (19), where vector [*u*, *v*, *φ*]^*T*^ represents absolute velocity in the local frame.

$$\begin{array}{c} \left[\begin{array}{c} \dot{x}\\ \dot{y}\\ \dot{\theta } \end{array}]=[\begin{array}{ccc} cos\theta & -sin\theta & 0\\ sin\theta & cos\theta & 0\\ 0 & 0 & 1 \end{array}][\begin{array}{c} u\\ v\\ \varphi \end{array}\right] \end{array}$$ (19)

The ground wheel interaction forces are represented by *F*_x**_ and *F*_y**_, where the second subscript represents front or rear wheel and 3^rd^ subscript represents left or right wheel. The dynamic equations of SSV in moving reference frame is given by (20)–(22).

$$\begin{array}{c} m\left(\dot{u}-\varphi v\right)=F_{xfl}+F_{xfr}+F_{xrl}+F_{xrr} \end{array}$$ (20)

$$\begin{array}{c} m\left(\dot{v}+\varphi u\right)=F_{yfl}+F_{yfr}+F_{yrl}+F_{yrr} \end{array}$$ (21)

$$\begin{array}{c} J\dot{\varphi }=-w_{l}F_{xrl}+w_{r}F_{xrr}-l_{r}F_{yrl}-l_{r}F_{yrr}-w_{l}F_{xfl}+w_{r}F_{xfr}+l_{f}F_{yfl}+l_{f}F_{yfr} \end{array}$$ (22)

where m is the mass, $\dot{u}$, $\dot{v}$ and $\dot{\varphi }$ are the longitudinal, lateral and yaw accelerations respectively. J is the vehicle’s moment of inertia while width and length are denoted by w and l respectively. The subscripts rl, rr, fl and fr represent the rear left, rear right, front left and front right respectively. Here, we also include the effects of the mass transfer between the wheels due to lateral and longitudinal accelerations and ground slope [32].

Moreover, due to the small size and weight of the SSV and stiffness of the suspension system, roll and pitch motions are neglected. Despite that, it is important to model the front/rear and left/right load transfers, due to lateral/longitudinal accelerations or due to ground slope, that affect the load distribution on the wheels and thus ground friction. Here, the vertical load on the wheel is represented by *F*_z**_, which includes the static load and load transfer. Thus, wheel load *F*_z**_ is calculated as follows [32]:

$$\begin{array}{c} F_{zfl}=F_{z}^{f,\;\text{static}\;}-\Delta F_{z}^{y,f}-\Delta F_{z}^{z},F_{zfr}=F_{z}^{f,\;\text{static}}+\Delta F_{z}^{y,f}-\Delta F_{z}^{z},F_{zrl}=F_{z}^{r,\;\text{static}\;}-\Delta F_{z}^{y,r}+\Delta F_{z}^{x},F_{zrr}=F_{z}^{r,\;\text{static}\;}+\Delta F_{z}^{y,r}+\Delta F_{z}^{x} \end{array}$$

Where:

$$\begin{array}{c} {\;F}_{z}^{f,\;\text{static}\;}=\frac{mg_{z}l_{f}}{2l},\;F_{z}^{r,\;\text{static}\;}=\frac{mg_{z}l_{r}}{2l}and:\Delta F_{z}^{y,f}=\frac{mhl_{f}}{wl}a_{x},\;\Delta F_{z}^{y,r}=\frac{mhl_{r}}{wl}a_{x},\;\Delta F_{z}^{x}=\frac{mh}{2l}a_{y} \end{array}$$

The effect of height h is also included in the above analysis. Here, $a_{x}=\dot{u}+g_{x}$ and $a_{y}=\dot{v}+g_{y}$. Where, *g*_*x*_, *g*_*y*_ and *g*_*z*_ refer to the components of the gravity acceleration vector g expressed in the vehicle local reference frame. Depending on the conditions stated above, the forces *F*_x**_ and *F*_y**_ while considering the effects of mass transfer on wheels, are utilized in (20)–(23). The spin dynamics of the wheel is computed by considering the balance of wheel torques.

$$\begin{array}{c} \begin{array}{cc} J_{w}{\dot{\omega }}_{fl} & =\tau_{fl}-RF_{xfl}\\ J_{w}{\dot{\omega }}_{fr} & =\tau_{fr}-RF_{xfr}\\ J_{w}{\dot{\omega }}_{rl} & =\tau_{rl}-RF_{xrl}\\ J_{w}{\dot{\omega }}_{rr} & =\tau_{rr}-RF_{xrr} \end{array} \end{array}$$ (23)

where *J*_*w*_ is the wheel’s moment of inertia, ${\dot{\omega }}_{**}$ is the vehicle angular acceleration, R is the effective radius and *τ* is the torque of the wheel.

### TM-easy (TME) tire model parameters for P-3AT robot

In [32], the pitch and roll motion is not considered. The moment of inertia of P-3AT is calculated about Z axis attached to the robot CG. The location of the CG of the P-3AT does not coincide with the geometrical centre but instead is displaced by adding of robotic arm at different location. This causes different mass distribution on the four wheels of the robot. The irregular mass distribution results in the change in load distribution on the wheel. The parameters of P-3AT robot with displaced CG are calculated experimentally from the P-3AT and the data is given in Table 1.

**Table 1:** Parameters of P-3AT robots for displaced CG.

Parameters	Magnitude	Units
P3AT Robot mass including payload	35.83	kg
Polar Moment of Inertia of robot (Z axis)	0.4101	kg-m^2^
CG location (width × length × height)	0.195 × 0.138 × 0.18	m
Front left wheel moment of inertia	0.0603	kg-m^2^
Rear left wheel moment of inertia	0.0603	kg-m^2^
Front right wheel moment of inertia	0.0603	kg-m^2^
Rear right wheel moment of inertia	0.0603	kg-m^2^
Robot’s dimensions (width × length)	0.395 × 0.26	m
Tire radius	0.11	m

In TME tire model, the maximum longitudinal and lateral forces, full sliding values and their corresponding slips are dependent upon the coefficient of friction and vertical inertial load of P3AT. In this paper, both the longitudinal and lateral coefficient of friction (*μ*_*x*_ and *μ*_*y*_) is changed by considering different surfaces and tire model parameters. Tire parameters in this case are considered for different real surfaces, hence, it leads to a real-world SSV model. The data in Table 2 is calculated experimentally from a laboratory robot while the Tables 3–7 is calculated directly from Table 2 according to the conversion formulas mentioned in the “influence of friction coefficient on tire parameters” section. Here, it is worth mentioning that the robot parameters are defined in [33], according to which, the robot can carry a maximum payload of about 40kg. Since the parameters of the robot were measured experimentally in the laboratory in the present work, so our measured parameters may not be exactly same as in [33], since, small measurement inaccuracy may exist. However, we are proposing robust controller which can compensate parametric and measurement uncertainties. The tire model parameters for different sets of coefficient of friction values and displaced CG are tabulated in Tables 2–7.

**Table 2:** Tire model parameters for *μ*_*x*_ = 0.5 and *μ*_*y*_ = 0.4.

Constants	Values (LF/LR/RF/RR)
$dF_{x}^{0}$	5.0448/3.6685/4.5894/4.8171N
$dF_{y}^{0}$	6.3808/4.64/5.8048/6.0928 N
$S_{y}^{M}$	23.8%
$S_{x}^{M}$	300%
$F_{y}^{M}$	39.88/29/36.28/38.08 N
$F_{x}^{M}$	50.448 /36.6850 /45.894 /48.171 N
$F_{y}^{f}$	31.1024 /22.617 /28.295 /29.699 N
$F_{x}^{f}$	37.847 /27.522 /34.431 /36.1391 N
$S_{y}^{f}$	300%
$S_{x}^{f}$	500%

**Table 3:** Tire model parameters for *μ*_*x*_ = 0.4 and *μ*_*y*_ = 0.2.

Constants	Values (LF/LR/RF/RR)
$dF_{x}^{0}$	5.044 /3.668 /4.589 /4.817 N
$dF_{y}^{0}$	6.380 /4.640 /5.804 /6.092 N
$S_{y}^{M}$	11.9%
$S_{x}^{M}$	23.7%
$F_{y}^{M}$	19.94/14.5/18.14/19.04 N
$F_{x}^{M}$	39.88/29/36.28/38.08 N
$F_{y}^{f}$	15.5512/11.3085/14.14735/14.8493 N
$F_{x}^{f}$	29.9186/21.7564/27.2180/28.5685 N
$S_{y}^{f}$	150%
$S_{x}^{f}$	395.2%

**Table 4:** Tire model parameters for *μ*_*x*_ = 0.6 and *μ*_*y*_ = 0.4.

Constants	Values (LF/LR/RF/RR)
$dF_{x}^{0}$	5.044 /3.668 /4.589 /4.817 N
$dF_{y}^{0}$	6.380 /4.640 /5.804/6.092 N
$S_{y}^{M}$	23.8%
$S_{x}^{M}$	35.5%
$F_{y}^{M}$	39.880 /29.1 /36.280 /38.08 N
$F_{x}^{M}$	59.82/43.5/54.42/57.12 N
$F_{y}^{f}$	31.1024/22.6171/28.2947/29.6986 N
$F_{x}^{f}$	44.8778/32.6347/40.8270/42.8527 N
$S_{y}^{f}$	300%
$S_{x}^{f}$	592.9%

**Table 5:** Tire model parameters for *μ*_*x*_ = 0.7 and *μ*_*y*_ = 0.5.

Constants	Values (LF/LR/RF/RR)
$dF_{x}^{0}$	5.044 /3.668 /4.589 /4.817 N
$dF_{y}^{0}$	6.380 /4.64/5.804 /6.092 N
$S_{y}^{M}$	29.75%
$S_{x}^{M}$	41.5%
$F_{y}^{M}$	49.85/36.25/45.35/47.6 N
$F_{x}^{M}$	69.79/50.75/63.49/66.64 N
$F_{y}^{f}$	38.878/28.2714/35.3684/37.1232 N
$F_{x}^{f}$	52.3575/38.0738/47.6315/49.9948 N
$S_{y}^{f}$	375%
$S_{x}^{f}$	691.7%

**Table 6:** Tire model parameters for *μ*_*x*_ = 0.9 and *μ*_*y*_ = 0.5.

Constants	Values (LF/LR/RF/RR)
$dF_{x}^{0}$	5.044/3.668/4.589/4.817N
$dF_{y}^{0}$	6.380/4.64/5.804/6.092 N
$S_{y}^{M}$	29.75%
$S_{x}^{M}$	53.3%
$F_{y}^{M}$	49.85/36.25/45.35/47.6 N
$F_{x}^{M}$	89.73/65.25/81.63/85.68 N
$F_{y}^{f}$	38.878/28.2714/35.3684/37.1232 N
$F_{x}^{f}$	67.3168/48.9520/61.2405/64.2790 N
$S_{y}^{f}$	375%
$S_{x}^{f}$	889.3%

**Table 7:** Tire model parameters for *μ*_*x*_ = 0.3 and *μ*_*y*_ = 0.2.

Constants	Values (LF/LR/RF/RR)
$dF_{x}^{0}$	5.044 /3.668 /4.589 /4.817 N
$dF_{y}^{0}$	6.380 /4.640 /5.804 /6.092 N
$S_{y}^{M}$	11.9%
$S_{x}^{M}$	17.76%
$F_{y}^{M}$	19.94/14.5/18.14/19.04 N
$F_{x}^{M}$	29.91/21.75/27.21/28.56 N
$F_{y}^{f}$	15.5512/11.3085/14.14735/14.8493 N
$F_{x}^{f}$	22.43/16.31/20.41/21.42 N
$S_{y}^{f}$	150%
$S_{x}^{f}$	296.4%

The parameters will emulate the behaviour of wet surfaces having low friction coefficient and dry surfaces with high friction coefficient.

### Four-wheel SSV model implementation

**Fig 6:**
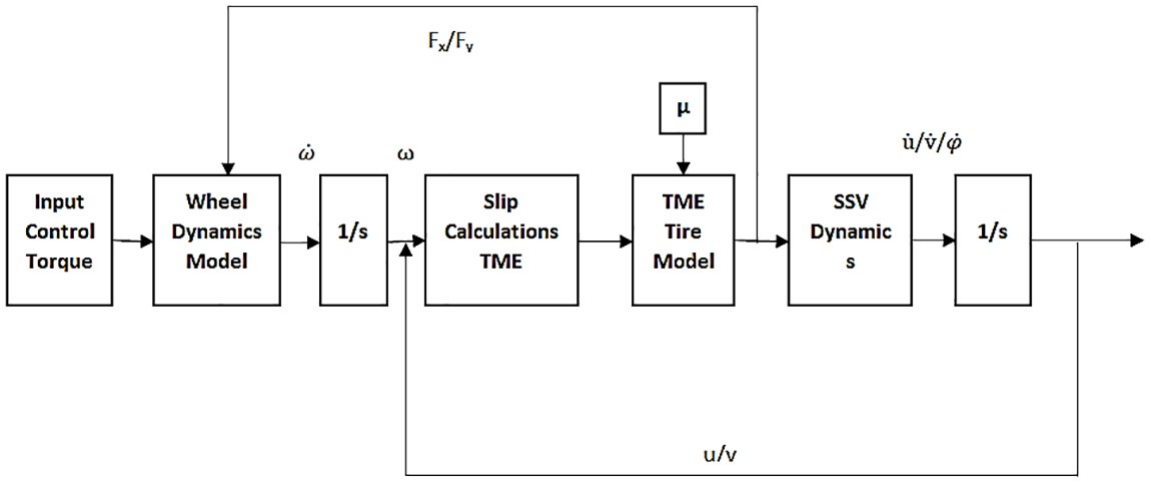
Block diagram of four-wheel SSV.

TM-easy tire model and the longitudinal and lateral forces are utilized to implement and calculate SSV dynamics. The equations of the parameters required for tire forces and the slips are implemented in MATLAB/Simulink Version 2020. Fig 6 illustrates the block diagram of four-wheel SSV implementation in Simulink. The SSV model receives control torque as an input from the feedback controller, which controls wheel torques. The wheel model calculates angular velocity from angular acceleration and generates feedback comprising of force components. Slip variables are calculated which are integrated to TM-Easy tire model to determine the generalized force components. Using SSV dynamics, longitudinal, lateral and yaw accelerations are computed which are integrated back as velocities for slip calculations. SSV dynamics block uses the outputs from tire model and vehicle model to simulate the SSV behavior. The TME tire model parameters and the robot parameters are individually incorporated into SSV model block. The model is simulated individually for different TME tire model parameters. The longitudinal velocities increase for few seconds and then get stabilized. The lateral velocity changes slightly because the vehicle is not skidding and it is trying to move in a straight line.

## Fuzzy sliding mode control architecture

**Fig 7:**
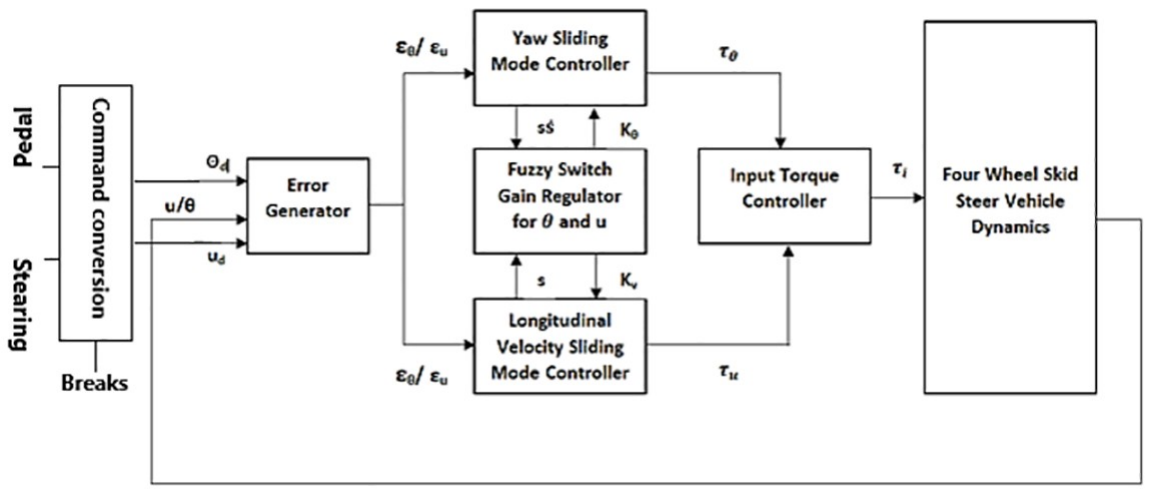
Proposed control architecture.

It is assumed that there are discrepancies between the actual mathematical model and the one that is developed for the controller design. However, these discrepancies are compensated by a robust method known as variable structure control system (VSCS). The procedure to develop SMC based law involves the controller design for yaw (*θ*) as well as for velocity (u). Fuzzy logic is adopted for switching gain regulations of the high constant gains for the discontinuous control part. Two additional parameters *τ*_*u*_ and *τ*_*θ*_ are introduced for the control of velocity and yaw respectively. *τ*_*u*_ is the velocity control torque produced by the longitudinal velocity controller and is equally applied to all the four wheels. *τ*_*θ*_ is the torque generated by yaw SMC and is added to and subtracted from the left and right wheels. The control scheme is shown in Fig 7. As shown in the Figure, the reference yaw angle and longitudinal velocity commands are calculated using command conversion block. The inputs to the command conversion block are pedal, steering and braking function. The command conversion block reads the sensor data from pedal and steering, and converts it into the reference commands while ensuring calibration.

The yaw and velocity control torques are calculated using (24)–(26), where N represents the total number of tires and the torque is divided equally among the tires. The yaw moment dynamics is given by (26).

$$\begin{array}{c} \tau_{u}=\sum_{i=1}^{N}\tau_{i} \end{array}$$ (24)

$$\begin{array}{c} \tau_{\theta }=\sum_{i=1}^{N}-\frac{w_{i}}{R}\tau_{i} \end{array}$$ (25)

$$\begin{array}{c} J\dot{\varphi }=\sum_{i=1}^{N}\left(-w_{i}F_{xi}+l_{i}F_{yi}\right) \end{array}$$ (26)

### Yaw sliding mode controller

The yaw moment of the SSV is expressed based on wheel dynamics. The input wheel torque expressed in (27) is obtained by generalizing the equations of the spin dynamics of the wheels shown in (23). *τ*_*i*_ is substituted into *τ*_*θ*_ in (25) to obtain (28).

$$\begin{array}{c} \tau_{i}=J_{\omega }{\dot{\omega }}_{i}+{RF}_{xi} \end{array}$$ (27)

$$\begin{array}{c} \tau_{\theta }=\sum_{i=1}^{N}-\frac{w_{i}}{R}\left(J_{\omega }{\dot{\omega }}_{i}+{RF}_{xi}\right) \end{array}$$ (28)

Re-arranging (26), we get the following expression.

$$\begin{array}{c} J\dot{\varphi }-\sum_{i=1}^{N}l_{i}F_{yi}=\sum_{i=1}^{N}-w_{i}F_{xi} \end{array}$$ (29)

By combining (28) and (29), one obtains (30).

$$\begin{array}{c} \dot{\varphi }=\lambda \tau_{\theta }+\lambda_{\theta }\dot{\omega }+D_{\theta }F_{y} \end{array}$$ (30)

Terms and parameters of (30) are expressed below. The vector product of these terms according to (30) will give the desired summation.

$$\begin{array}{c} \begin{array}{cc} \lambda & =\frac{1}{J}\\ {\;\;\;\;\;\;\;\;\;\;\;\;\;\;\;\;\;\;\lambda }_{\theta } & =\frac{J_{\omega }}{JR}\left[\ldots .w_{i}\ldots .\right]\\ D_{\theta } & =\frac{1}{J}\left[\ldots .l_{i}\ldots .\right]\\ \dot{\omega } & ={\left[\ldots .{\dot{\omega }}_{i}\ldots .\right]}^{T}\\ F_{y} & =[\ldots F_{yi}{\ldots ]}^{T} \end{array} \end{array}$$ (31)

Considering *c*_*dθ*_ as a control law and by considering uncertainties $n(\theta ,\varphi ,\dot{\varphi )}$ in the dynamic equation, the following expressions are introduced:

$$\begin{array}{c} c_{d\theta }={\dot{\varphi }}_{d}+K_{p}^{\theta }\varepsilon_{\theta }+K_{d}^{\theta }{\dot{\varepsilon }}_{\theta }+\sigma_{\theta } \end{array}$$ (32)

$$\begin{array}{c} \dot{\varphi }=c_{d\theta }-n\left(\theta ,\varphi ,\dot{\varphi }\right) \end{array}$$ (33)

where the derived yaw acceleration is ${\dot{\varphi }}_{d}$ and the yaw error is *ϵ*_*θ*_ = *θ*_*d*_ − *θ*. The transient response of the system is controlled by the constants $K_{p}^{\theta }$ and $K_{d}^{\theta }$, and SMC law is represented by *σ*_*θ*_ in (32). The double derivative of *ε*_*θ*_ formulates the error state equation. By combining (32) and (33) to formulate ${\ddot{\varepsilon }}_{\theta }$ results in (34). The state vector is defined as $x={\left[\epsilon_{\theta },\;{\dot{\varepsilon }}_{\theta }\right]}^{T}$ and is presented as follows:

$$\begin{array}{c} {\ddot{\varepsilon }}_{\theta }={\dot{\varphi }}_{d}-\dot{\varphi }=-K_{p}^{\theta }\varepsilon_{\theta }-K_{d}^{\theta }{\dot{\varepsilon }}_{\theta }+n-\sigma_{\theta } \end{array}$$ (34)

$$\begin{array}{c} \dot{x}=Ax+B(n-\sigma_{\theta }) \end{array}$$ (35)

where the matrices A and B are shown as follows:

A=(01-Kpθ-Kdθ),B=(01)

For tracking the desired yaw angle and to prove the closed loop stability, the Lyapunov candidate function is chosen as follows: *V* = *x*^*T*^ *Px*, where P is positive definite symmetric matrix. The state x = 0 is stable if the criteria given below is satisfied.

$$\begin{array}{c} V(0)=0\;;\forall (x)=0\;\;\;while\;\;V(x)>0\;and\;\dot{V}(x)<0\;;\;\forall (x)\neq 0\; \end{array}$$ (36)

By finding the derivative of Lyapunov candidate function, (37) is obtained. Substituting (35) in (37) yields (38).

$$\begin{array}{c} \dot{V}(x)=s\dot{s}=\dot{x}Px+x^{T}P\dot{x} \end{array}$$ (37)

$$\begin{array}{c} \dot{V}(x)=(x^{T}A^{T}+nB^{T}-\sigma_{\theta }B^{T})Px+x^{T}P\left(Ax+Bn-{B\sigma }_{\theta }\right) \end{array}$$ (38)

The switching surface *s* = *B*^*T*^ *Px* is considered as scalar, hence, *B*^*T*^ *Px* = *x*^*T*^ *PB*. Using this relationship, (39) is obtained. where P is computed by (40), which is a Lyapunov equation. Finally, including (40) in (39), we get (41).

$$\begin{array}{c} \dot{V}=-x^{T}\left(A^{T}P+PA\right)x+2x^{T}PB\left(n-\sigma_{\theta }\right) \end{array}$$ (39)

$$\begin{array}{c} A^{T}P+PA=-Q_{l} \end{array}$$ (40)

$$\begin{array}{c} \dot{V}=-x^{T}Q_{l}x+2x^{T}PB\left(n-\sigma_{\theta }\right) \end{array}$$ (41)

In order to maintain stability, $\dot{V}$ should be negative definite. If x lies in the window of *B*^*T*^ *P*, the second term in (41) vanishes while the first term is negative. The second term has to be very small inside the boundary R. The variable s represents the sliding surface ideally when s = 0. The error state vector x is zero if s is zero. SMC law is suggested by the relay function of (42).

$$\begin{array}{c} \sigma_{\theta }=\rho_{u}\frac{s}{\left|s\right|} \end{array}$$ (42)

The norms s and *ρ*_*u*_ are positive scalars where *ρ*_*u*_ is to be large enough for stabilization.

### Fuzzy switch gain regulator for yaw angle

For yaw angle and longitudinal velocity controller, fuzzy switch gain regulator (FSGR) is calculated for the SMC terms. Fuzzy logic is used as a switching gain regulator for *ρ*_*u*_ term in (42). Depending on $s\dot{s}$, fuzzy logic adjusts *ρ*_*u*_. The existing condition for sliding mode is given by $s\dot{s}<0$, and if this equation is satisfied, it means that the states of the system are on sliding manifold. The effect of uncertainties must be removed by proper selection of the gain *ρ*_*u*_. The sliding mode existence condition is guaranteed by following fuzzy rule.

*ρ*_*u*_ must be increased If $s\dot{s}>0$
*ρ*_*u*_ must be decreased If $s\dot{s}\;<0$

Based on $s\dot{s}$, the change in sliding term gain is Δ*ρ*_*u*_. It controls the transient response on the sliding surfaces by reduction of tracking errors. For the yaw angle, switching gain regulator input and output, the fuzzy sets are calculated from the above rules; the input $s\dot{s}$ and the output Δ*ρ*_*u*_ are expressed as follows.

$s\dot{s}=\{\text{NB}\;\text{NM}\;\text{Z}\;\text{PM}\;\text{PB}\}$

Δ*ρ*_*u*_ = {NB NM Z PM PB}

where NB represents negative big, NM is negative medium and Z represents zero. Similarly PM represents positive medium and PB is positive big.

**Fig 8:**
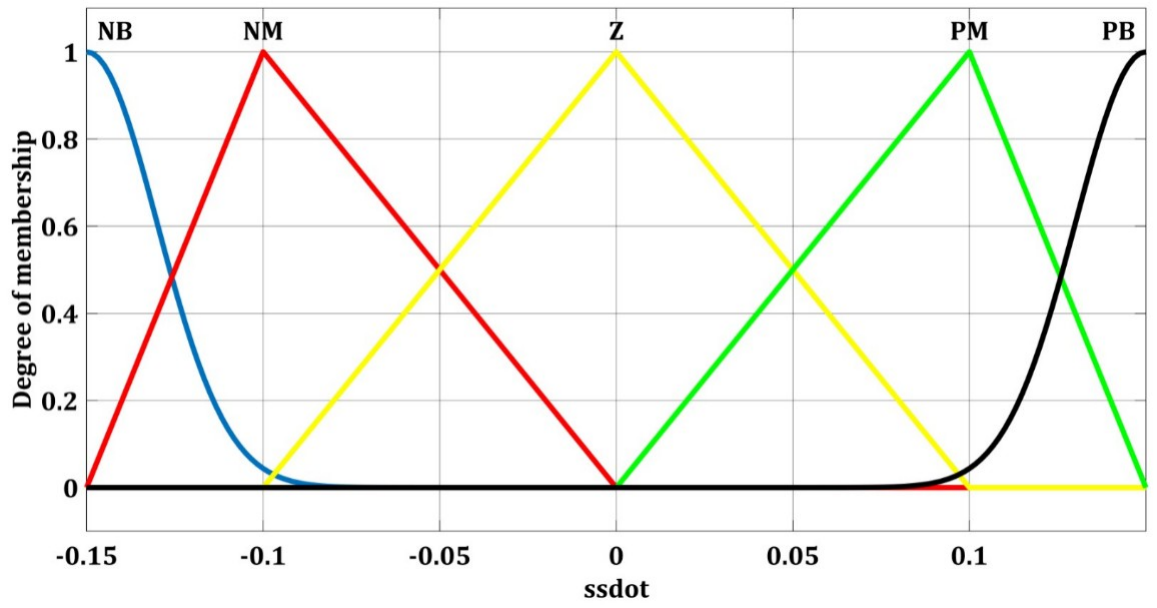
Fuzzy membership function for yaw with $s\dot{s}$.

**Fig 9:**
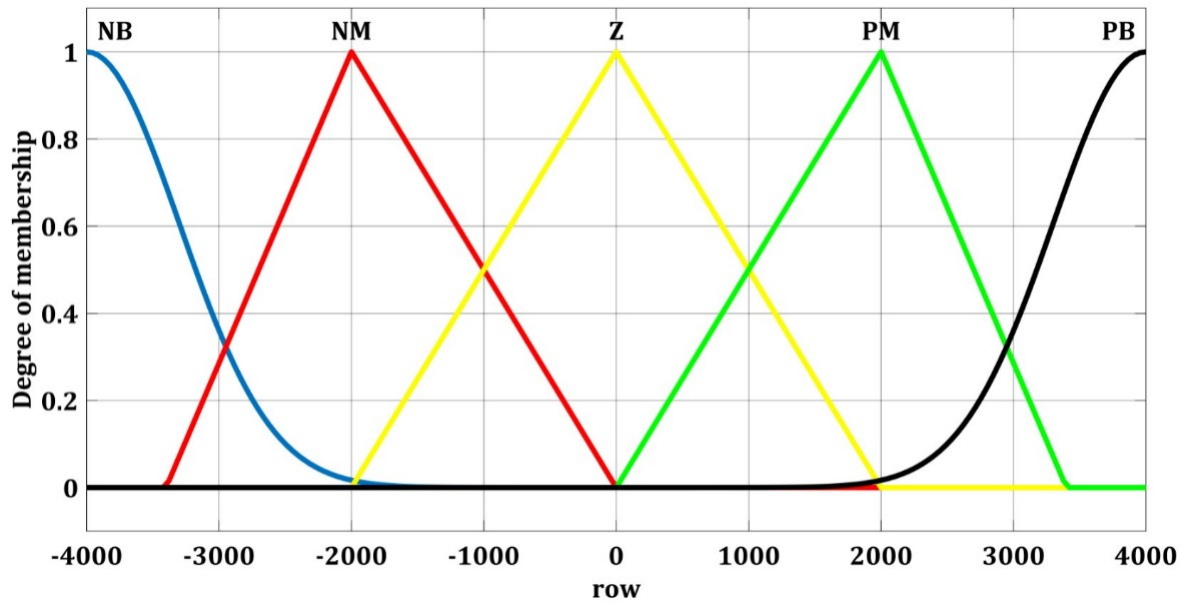
Fuzzy membership functions for yaw with Δ*ρ*_*u*_.

Fuzzy switching gain regulator membership functions of yaw angle for the input $s\dot{s}$ and output Δ*ρ*_*u*_ are shown in Figs 8 and 9 respectively. These five membership functions correspond to five sets.

Fuzzy rules picked for the yaw angle FSGR are given as follows:

R1: Δ*ρ*_*u*_ is PB when $s\dot{s}$ is PB
R2: Δ*ρ*_*u*_ is PM when $s\dot{s}$ is PM
R3: Δ*ρ*_*u*_ is Z when $s\dot{s}$ is Z
R4: Δ*ρ*_*u*_ is NM when $s\dot{s}$ is NM
R5: Δ*ρ*_*u*_ is NB when $s\dot{s}$ is NB

Eq (43) is used for the estimation of the super bound of ${\hat{K}}_{\theta }(t)$ using the integral method, where *G*_*θ*_ is the proportionality coefficient. ${\hat{K}}_{\theta }(t)$ is used in the equation of global law instead of *ρ*_*u*_. The FSGR adjusts to the stability requirement on $s\dot{s}$ as the system reaches sliding surface and changes according to the error. The online tuning of FSGR reduces chattering phenomena.

$$\begin{array}{c} \hat{K_{\theta }}(t)=G_{\theta }\int_{0}^{t}\Delta \rho_{u}dt \end{array}$$ (43)

$$\begin{array}{c} s(n\left(t\right)-\sigma_{\theta })=sn(t)-K(t)\frac{s^{2}}{\left|s\right|}=sn(t)-\hat{K_{\theta }}(t)|s|\leq |s|(|n(t)|-\hat{K_{\theta }}(t)) \end{array}$$ (44)

### Longitudinal velocity sliding mode control

The general equation of SSV dynamic model in longitudinal direction is given by (45). Modifying (45) yields (46).

$$\begin{array}{c} M(\dot{u}-\varphi v)=\sum_{i=1}^{N}F_{xi} \end{array}$$ (45)

$$\begin{array}{c} M(\dot{u}-\varphi v)=-\frac{J_{\omega }}{R}\sum_{i=1}^{N}{\dot{\omega }}_{i}+\frac{1}{R}\sum_{i=1}^{N}\tau_{i} \end{array}$$ (46)

Now, substituting the constants from (47) and (48) in (46) and then carrying further simplifications yields (49).

$$\begin{array}{c} \wedge_{u}=-\frac{J_{\omega }}{MR} \end{array}$$ (47)

$$\begin{array}{c} \varphi =\frac{1}{MR} \end{array}$$ (48)

$$\begin{array}{c} \dot{u}=\wedge_{u}\sum_{i=1}^{N}{\dot{\omega }}_{i}+\varphi \tau_{u}+\varphi v \end{array}$$ (49)

Let the control law is defined as *c*_*u*_, and let the uncertainties function is $m(u,\dot{u})$ then *c*_*u*_ is expressed as follows:

$$\begin{array}{c} \dot{u}=c_{u}-m(u,\dot{u}) \end{array}$$ (50)

where the control law for longitudinal direction is defined by (51) as.

$$\begin{array}{c} c_{u}={\dot{u}}_{d}+K_{p}^{u}\varepsilon_{u}+\sigma_{u} \end{array}$$ (51)

where the desired longitudinal acceleration is ${\dot{u}}_{d}$, *ϵ*_*u*_ = *u*_*d*_ − *u* is the velocity error in longitudinal direction, the constant $K_{p}^{u}$ controls the settling time of the closed loop system while *σ*_*u*_ is the SMC law. The choice of SMC law guarantees the stability of the system using Lyapunov candidate function.

### Fuzzy switch gain regulator for longitudinal velocity

**Fig 10:**
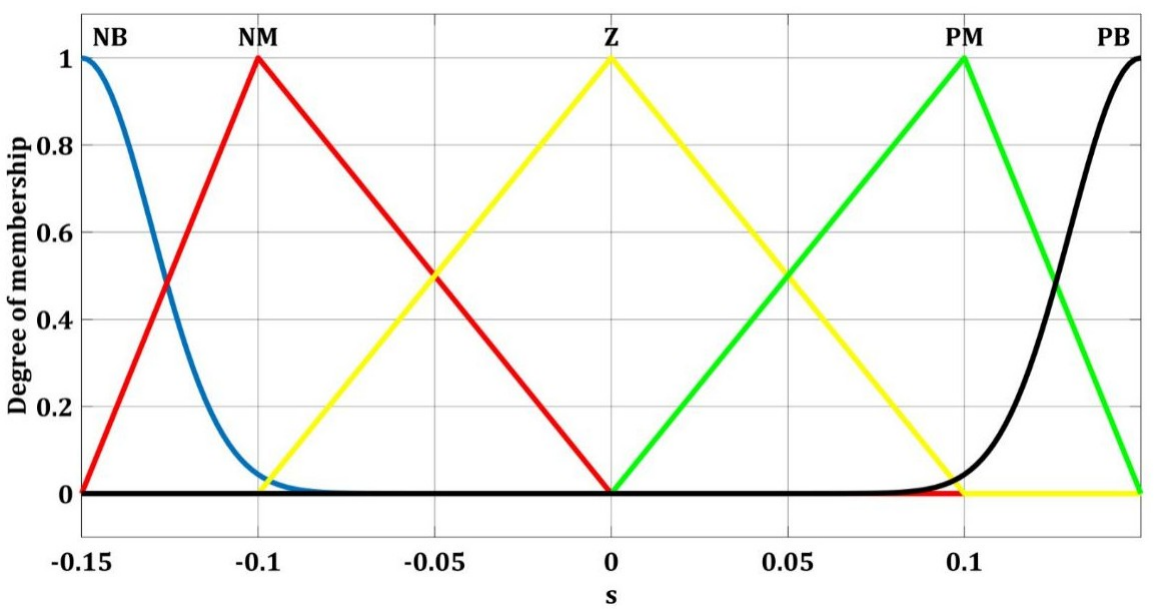
Fuzzy membership function for the longitudinal velocity with s.

**Fig 11:**
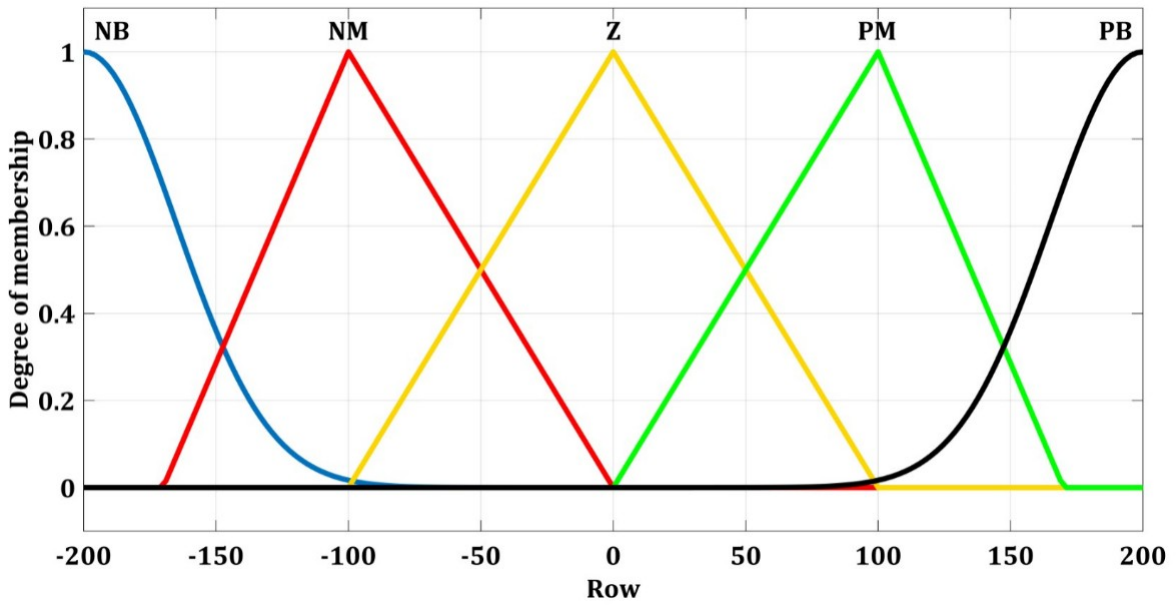
Fuzzy membership functions for longitudinal velocity with Δ*ρ*_*θ*_.

The fuzzy membership function of the longitudinal velocity FSGR with an input variable s and an output variable Δ*ρ*_*θ*_ are expressed in Figs 10 and 11.

All the rules and the input and output membership functions are the same as that of FSGR yaw angle controller. The torques *τ*_*u*_ and *τ*_*θ*_ are computed individually for each wheel by the velocity controller and the yaw angle controller respectively and are represented by (52).

$$\begin{array}{c} \begin{array}{cc} \tau_{u} & =\frac{1}{\varphi }\left({\dot{u}}_{d}+K_{p}^{u}\varepsilon_{u}+\rho_{u}\frac{\varepsilon_{u}}{\|\varepsilon_{u}\|+v_{u}}\right)\\ \tau_{\theta } & =\frac{1}{\lambda }\left(\dot{\emptyset_{d}}+K_{p}^{\theta }\varepsilon_{\theta }+K_{d}^{\theta }{\dot{\varepsilon }}_{\theta }+\rho_{\theta }\frac{B^{T}Px}{\|B^{T}Px\|+v_{\theta }}\right) \end{array} \end{array}$$ (52)

The applied torques on each wheel are calculated by (53) and (54) and expressed as follows:

$$\begin{array}{c} \tau_{fl}=\tau_{rl}=\tau_{u}-\frac{\tau_{\theta }}{2} \end{array}$$ (53)

$$\begin{array}{c} \tau_{fr}=\tau_{rr}=\tau_{u}+\frac{\tau_{\theta }}{2} \end{array}$$ (54)

### Fractional order yaw angle sliding mode control

In this section, fractional order yaw angle controller is formulated. A fractional operator is defined as *D*^*α*^ where *α* represents the order of fractional operator. Furthermore, when *α* is positive, *D*^*α*^ represents a fractional derivative, while when *α* is negative, *D*^−*α*^ represents a fractional integral. The fractional operator is approximated using an Oustaloup filter detailed below [34]:

$$\begin{array}{c} s^{\alpha }=K\prod_{i=-n}^{n}\;\;\;\frac{\left(1+\frac{s}{w_{z,i}}\right)}{\left(1+\frac{s}{w_{p,i}}\right)} \end{array}$$ (55)

Here, *α* is positive, K represents gain. *w*_*z*,*i*_ and *w*_*p*,*i*_ are defined as follows:

$$\begin{array}{c} \begin{array}{cc} w_{p,i} & =w_{b}{\left(\frac{w_{h}}{w_{b}}\right)}^{\frac{\left(i+n+0.5)(1+\alpha \right)}{2n+1}}\\ w_{z,i} & =w_{b}{\left(\frac{w_{h}}{w_{b}}\right)}^{\frac{\left(i+n+0.5)(1-\alpha \right)}{2n+1}} \end{array} \end{array}$$ (56)

Where *w*_*b*_ and *w*_*h*_ represent the lower and upper bounds of frequencies respectively. Now let us define a fractional order Lyapunov function for the yaw angle dynamics as follows:

$$\begin{array}{c} V_{\theta }=0.5D^{\alpha }{\dot{\varepsilon_{\theta }}}^{2}+0.5K_{P}D^{\alpha }{\varepsilon_{\theta }}^{2} \end{array}$$ (57)

The first derivative of (57) w.r.t. time yields the following expression.

$$\begin{array}{c} \dot{V_{\theta }}=D^{\alpha }\dot{\varepsilon_{\theta }}\ddot{\varepsilon_{\theta }}+K_{P}{D^{\alpha }\dot{\varepsilon_{\theta }}\varepsilon }_{\theta } \end{array}$$ (58)

Eq (58) is simplified as follows:

$$\begin{array}{c} \dot{V_{\theta }}=D^{\alpha }\dot{\varepsilon_{\theta }}\left(\ddot{\varepsilon_{\theta }}+K_{P}\varepsilon_{\theta }\right) \end{array}$$ (59)

By using *ε*_*θ*_, and by combining (30) with (59), one obtains the following relation:

$$\begin{array}{c} \dot{V_{\theta }}=D^{\alpha }\dot{\varepsilon_{\theta }}\left(\dot{\emptyset_{d}}-\lambda \tau_{\theta }-n+K_{P}\varepsilon_{\theta }\right) \end{array}$$ (60)

Here, n represents the disturbance term and it is equated as follows: ${n=\lambda }_{\theta }\dot{\omega }+D_{\theta }F_{y}$. From (60), the yaw controller is designed as follows:

$$\begin{array}{c} \tau_{\theta }=\frac{1}{\lambda }{[\dot{\emptyset_{d}}+K_{d}D}^{1-\alpha }\varepsilon_{\theta }+K_{P}\varepsilon_{\theta }+\rho_{\theta }D^{-\alpha }sgn\left(\dot{\varepsilon_{\theta }}\right)] \end{array}$$ (61)

Now by combining (61) with (60), the closed loop stability is ensured and is explained as follows:

$$\begin{array}{c} \dot{V_{\theta }}=-K_{d}{\dot{\varepsilon_{\theta }}}^{2}-n\dot{\varepsilon_{\theta }}-\rho_{\theta }\left|\dot{\varepsilon_{\theta }}\right| \end{array}$$ (62)

The first term is always negative when *K*_*d*_ > 0, the second term of (62), may be positive or negative depending on $\dot{\varepsilon_{\theta }}$, while the third term is also negative when *ρ*_*θ*_ > 0. Thus, $\dot{V_{\theta }}$ is also negative if the cumulative effect of $-K_{d}{\dot{\varepsilon_{\theta }}}^{2}-\rho_{\theta }\left|\dot{\varepsilon_{\theta }}\right|$ is more negative than the second term $-n\dot{\varepsilon_{\theta }}$.

**Note**: Comparing (61) with (52), it is clear that the proposed fractional order controller does not depend on the yaw rate, instead it includes a term *D*^1−*α*^ *ε*_*θ*_. With *α* < 1 (fractional orders), the term *D*^1−*α*^ *ε*_*θ*_ represents a fractional derivative of the yaw angle error. As mentioned in [29, 31], a fractional operator is robust to the measurement noise, thus yaw rate is no more required and the proposed controller can be formulated with the measured yaw angle. Moreover, in (61), a fractional integral is calculated around sgn(.) term and it will be helpful in smoothing the oscillations further.

### Fractional order longitudinal velocity sliding mode control

In order to formulate a fractional order controller for longitudinal velocity control of SSV, a fractional order Lyapunov function is defined as follows:

$$\begin{array}{c} V_{u}=0.5{D^{\alpha }\varepsilon }_{u} \end{array}$$ (63)

The first derivative of (63) w.r.t. time yields the following expression.

$$\begin{array}{c} \dot{V_{u}}={D^{\alpha }\dot{\varepsilon_{u}}\varepsilon }_{u} \end{array}$$ (64)

Using (49), Eq (64) is simplified as follows:

$$\begin{array}{c} \dot{V_{u}}=D^{\alpha }\varepsilon_{u}\left(\dot{u_{d}}-\emptyset \tau_{u}-m\right) \end{array}$$ (65)

Here, m represents the disturbance term and it is given as follows: $m=\wedge_{u}\sum_{i=1}^{N}{\dot{\omega }}_{i}+\varphi v$. From (65), the yaw controller is designed as follows:

$$\begin{array}{c} \tau_{u}=\frac{1}{\emptyset }{[\dot{u_{d}}+K_{p}D}^{-\alpha }\varepsilon_{u}+K_{P}\varepsilon_{\theta }+\rho_{u}D^{-\alpha }sgn\left(\varepsilon_{u}\right)] \end{array}$$ (66)

Now, by combining (66) with (65), the closed loop stability is ensured and is explained as follows:

$$\begin{array}{c} \dot{V_{u}}=-K_{p}{\varepsilon_{u}}^{2}-m\varepsilon_{u}-\rho_{u}\left|\varepsilon_{u}\right| \end{array}$$ (67)

The first term is always negative when *K*_*p*_ > 0, the second term of (67), may be positive or negative depending on *ε*_*u*_, while the third term is also negative when *ρ*_*u*_ > 0. Thus, $\dot{V_{u}}$ is also negative if the cumulative effect of −*K*_*p*_ *ε*_*u*_^2^ − *ρ*_*u*_|*ε*_*u*_| is more negative than the second term −*mε*_*u*_.

## Simulation results and comparative analysis

### Results and comparative study of classical SMC, FSMC and fractional order FSMC

The simulation analysis is conducted and the performance of FSMC and Fractional order FSMC algorithms is compared with the conventional SMC controller. The controllers are tested with friction coefficients of (0.506, 0.4). Moreover, the centre of gravity does not coincide with the geometric centre. The tuned SMC gains are given in Table 8. Since all the three variants of controllers are utilizing the same gains except some additional parameters are necessary to define for fractional order SMC. These parameters include the orders of fractional operator for *θ* and u loops. Hence, *α*_*θ*_ = *u*_*θ*_ = 0.95.

**Table 8:** Gains of SMC.

Gains	Magnitude
$K_{p}^{u}$	100
*ρ*_*u*_	50
*ρ*_*θ*_	300
*v*^*u*^ *and v*^*θ*^	0.015
*a and b*	1
$K_{p}^{\theta }$	6.25
$K_{d}^{\theta }$	5
*A*	0.65

**Fig 12:**
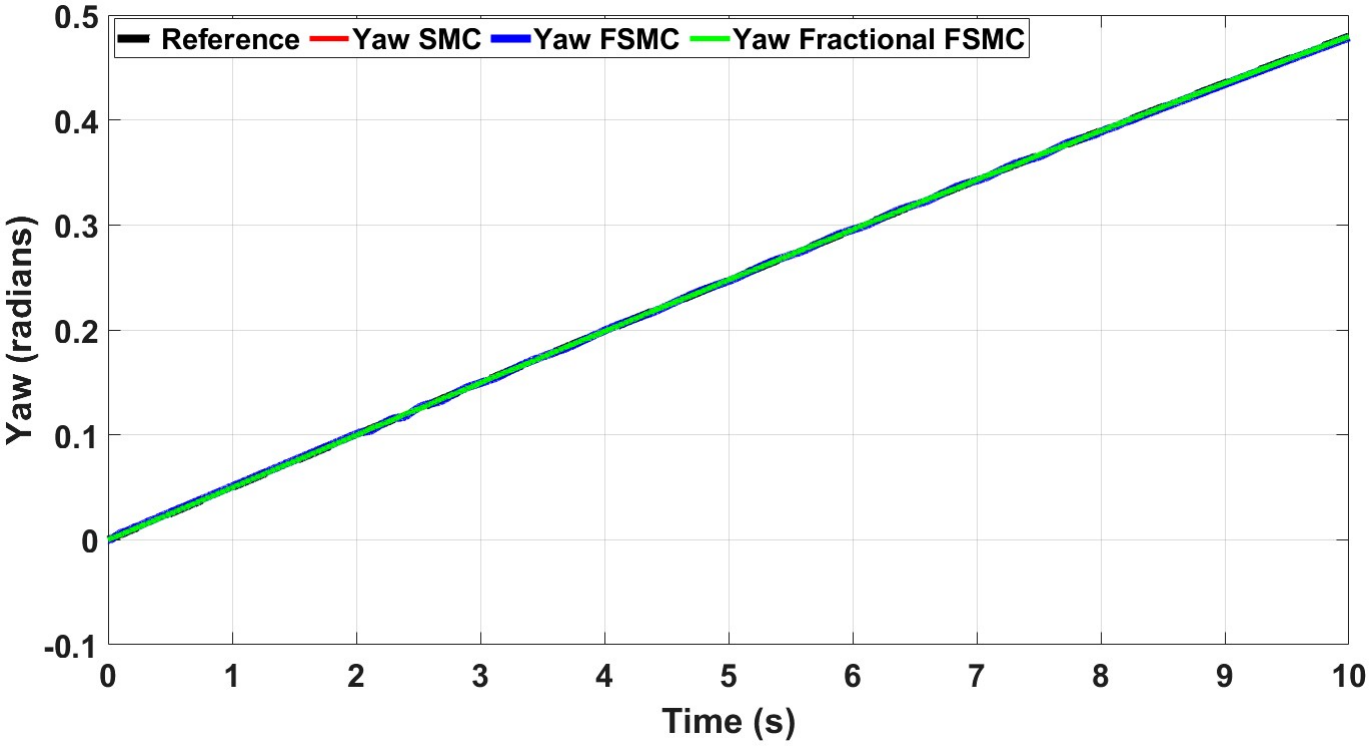
Yaw tracking comparison.

**Fig 13:**
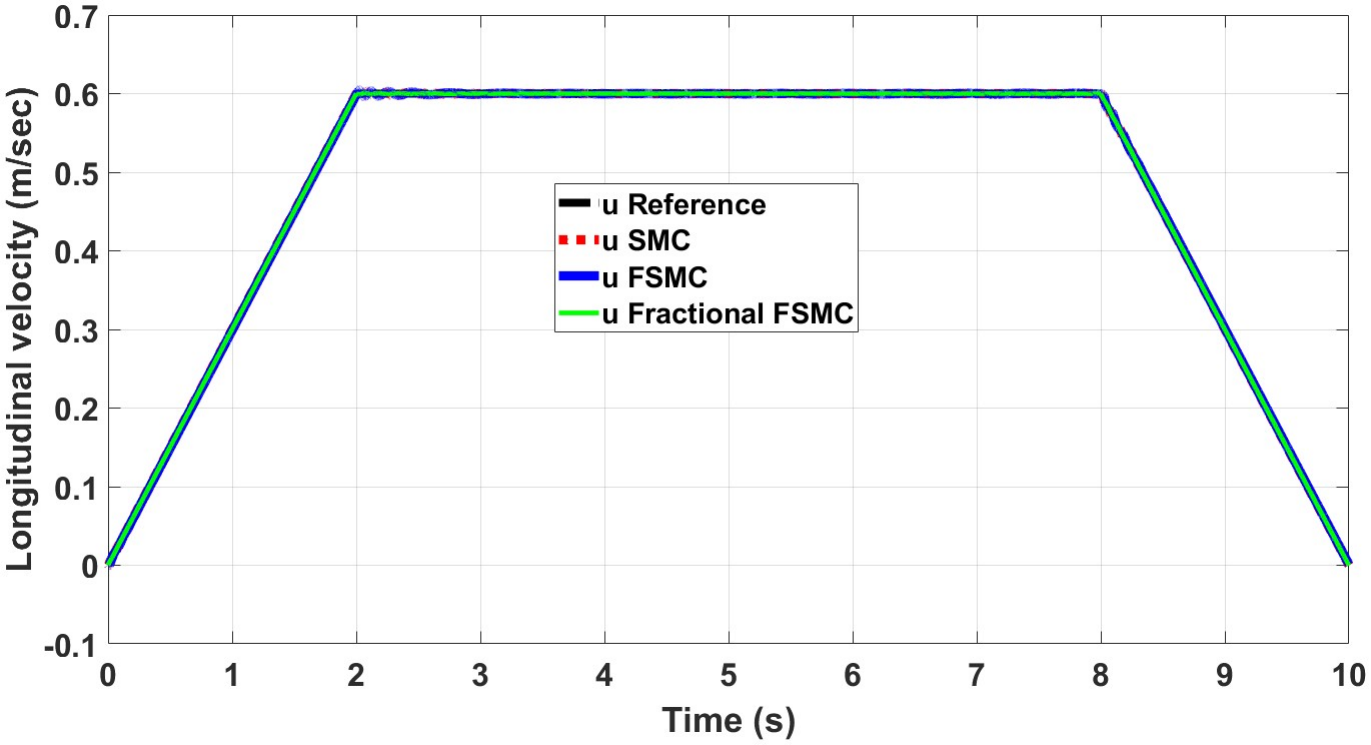
Longitudinal velocity tracking comparison.

Figs 12 and 13 show yaw and longitudinal velocity tracking response with SMC, FSMC and fractional order FSMC. In Figs 12 and 13, the SMC, FSMC and Fractional FSMC tracks the reference yaw and longitudinal velocity trajectories accurately with minimal errors. However, the FFSMC algorithm has better response than the FSMC and SMC algorithms. In Fig 12, the time response of all the controllers is very accurate and very minimal errors are observed. On the other hand in Fig 13, FFSMC algorithm ensures the minimal error as compared to the other 2 algorithms at different intervals.

**Fig 14:**
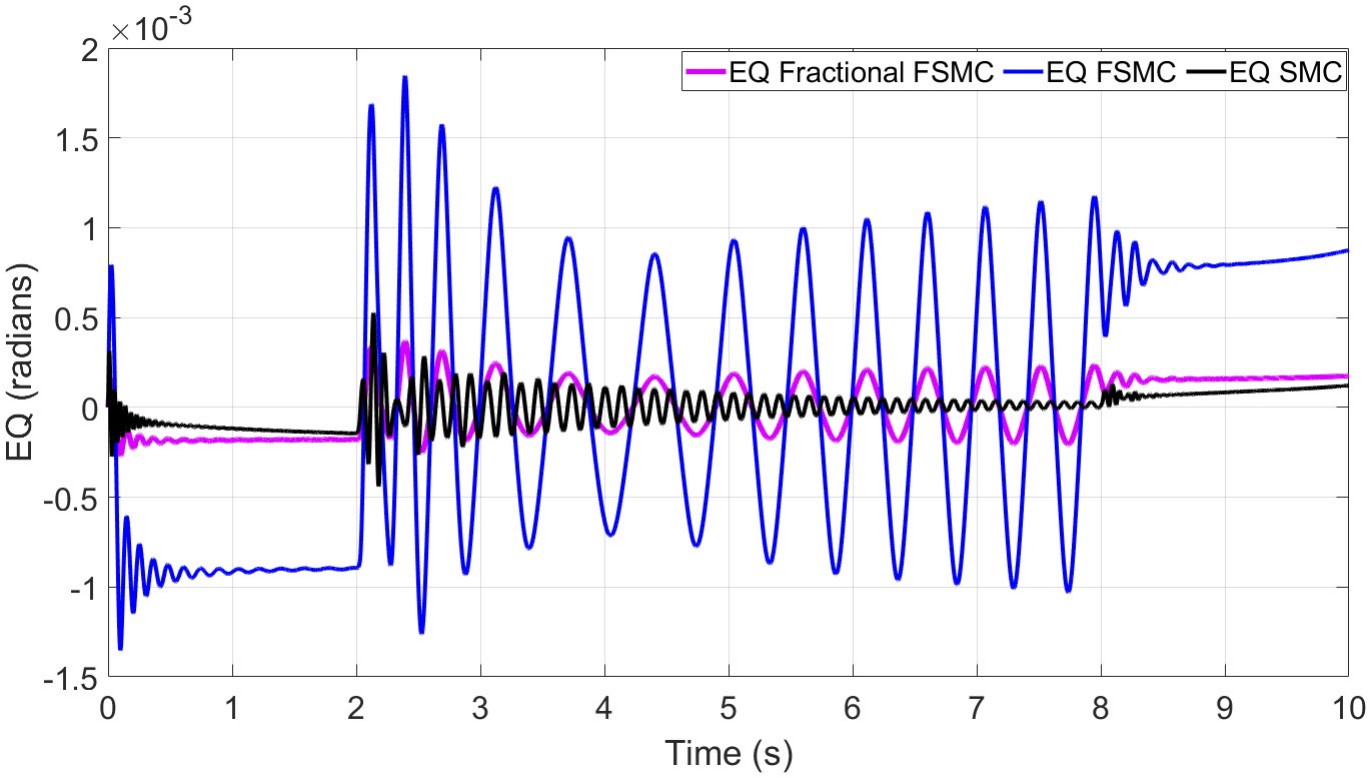
Comparison of yaw errors in case of SMC, FSMC and fractional FSMC.

**Fig 15:**
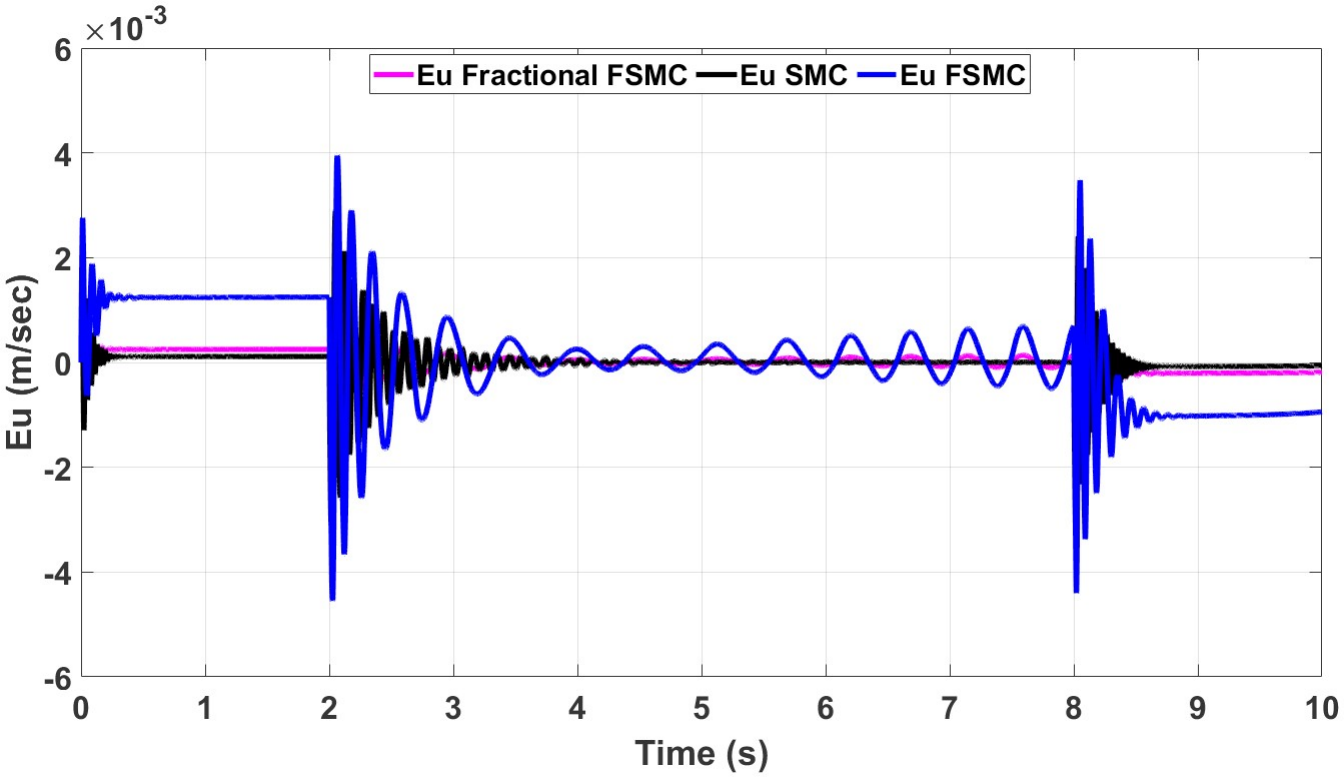
Comparison of velocity errors in case of SMC FSMC and fractional FSMC.

In order to have better readability of the responses given in Figs 12 and 13, the error plots for yaw and longitudinal velocity are given in Figs 14 and 15 respectively.

From the above plots, it is observed that the magnitude of error is large with FSMC based controller. The lowest errors are ensured using SMC, while with fractional order SMC, the magnitude of errors is comparable to SMC. Secondly, the frequency of oscillations reduces with both FSMC and fractional order FSMC. From Figs 14 and 15, and at time t = 2-4secs, the frequency of oscillations with SMC is twice as compared to the FSMC and fractional FSMC.

At start, the yaw error peaked at 0.0017 radians and at 2 and 8 seconds, it reduces to 0.0012 radians. At 2 and 8 seconds, there will be acceleration and deceleration respectively and it occurs at the curvature of the FSMC’s velocity profile. The FSMC error values are higher than those demonstrated by SMC. The yaw error of SMC at 0, 2 and 8 seconds are 0.001, 0.0005 and 0.0001 radians respectively. In the third case with fractional FSMC, the yaw error is peaked at 0.0025 radians at 2 seconds. The comparison is done in Table 9.

**Table 9:** Yaw and velocity errors comparison.

Time (sec)	Yaw Error (Radians)	Velocity Error (m/sec)
	**SMC Algorithm**	
0	0.001	2.2 × 10^−^3
2	0.0005	2.8 × 10^−^3
8	0.0001	2.2 × 10^−^3
	**FSMC Algorithm**	
0	0.0017	2.8 × 10^−^3
2	0.0012	4.4 × 10^−^3
8	0.0012	3.3 × 10^−^3
	**FFSMC Algorithm**	
0	0.0004	1 × 10^−^3
2	0.0003	1.2 × 10^−^3
8	0.0002	0.5 × 10^−^3

As discussed before, chattering phenomenon is observed in SMC with high oscillations during acceleration and deceleration. Hence, the chattering is minimized in case of FSMC and fractional FSMC based controllers.

**Fig 16:**
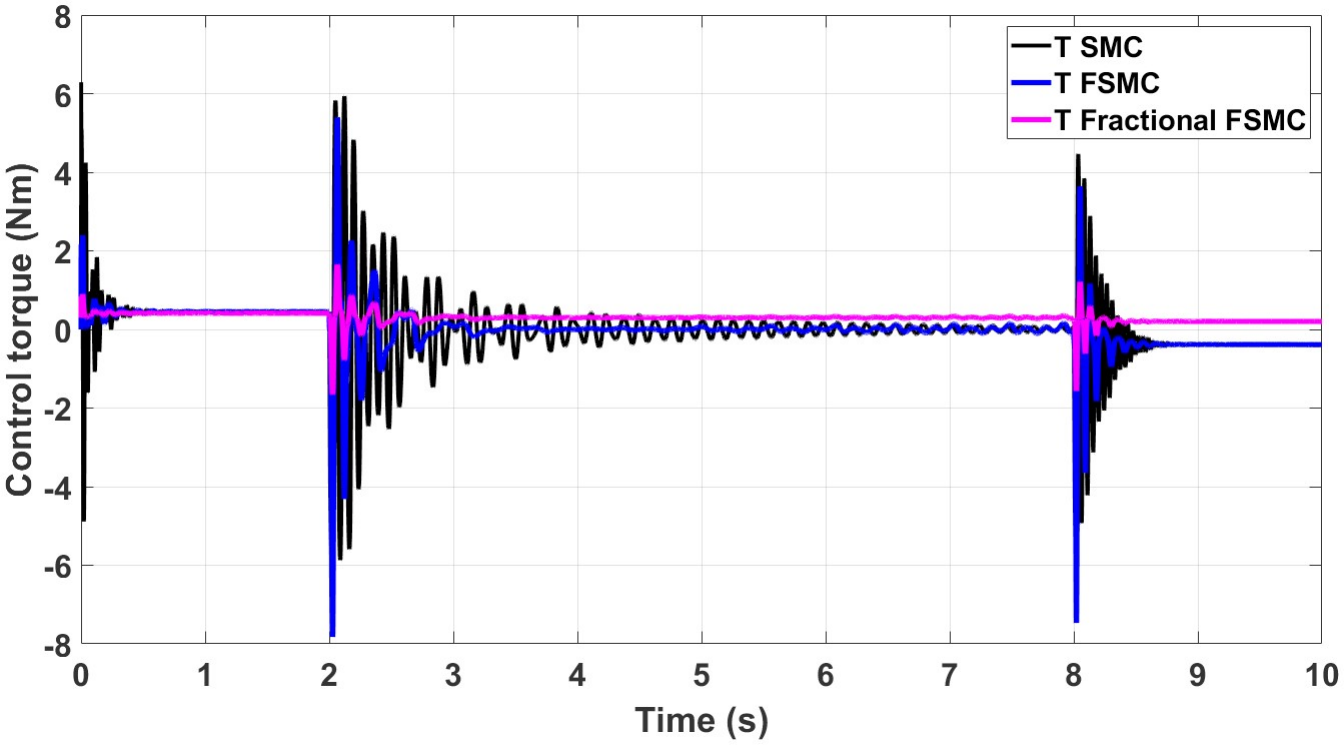
Control torque response generated by SMC, FSMC and fractional FSMC based laws.

**Fig 17:**
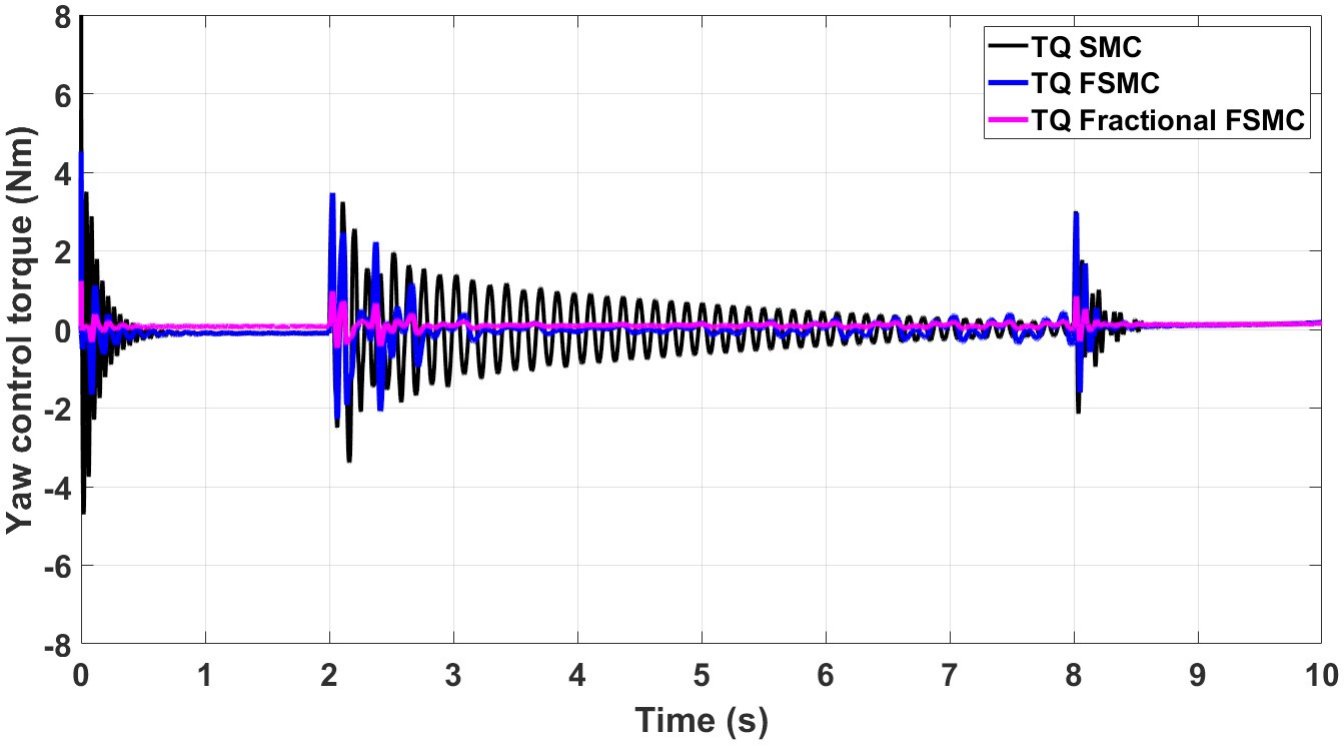
Yaw control torque response generated by SMC, FSMC and fractional FSMC based laws.

**Fig 18:**
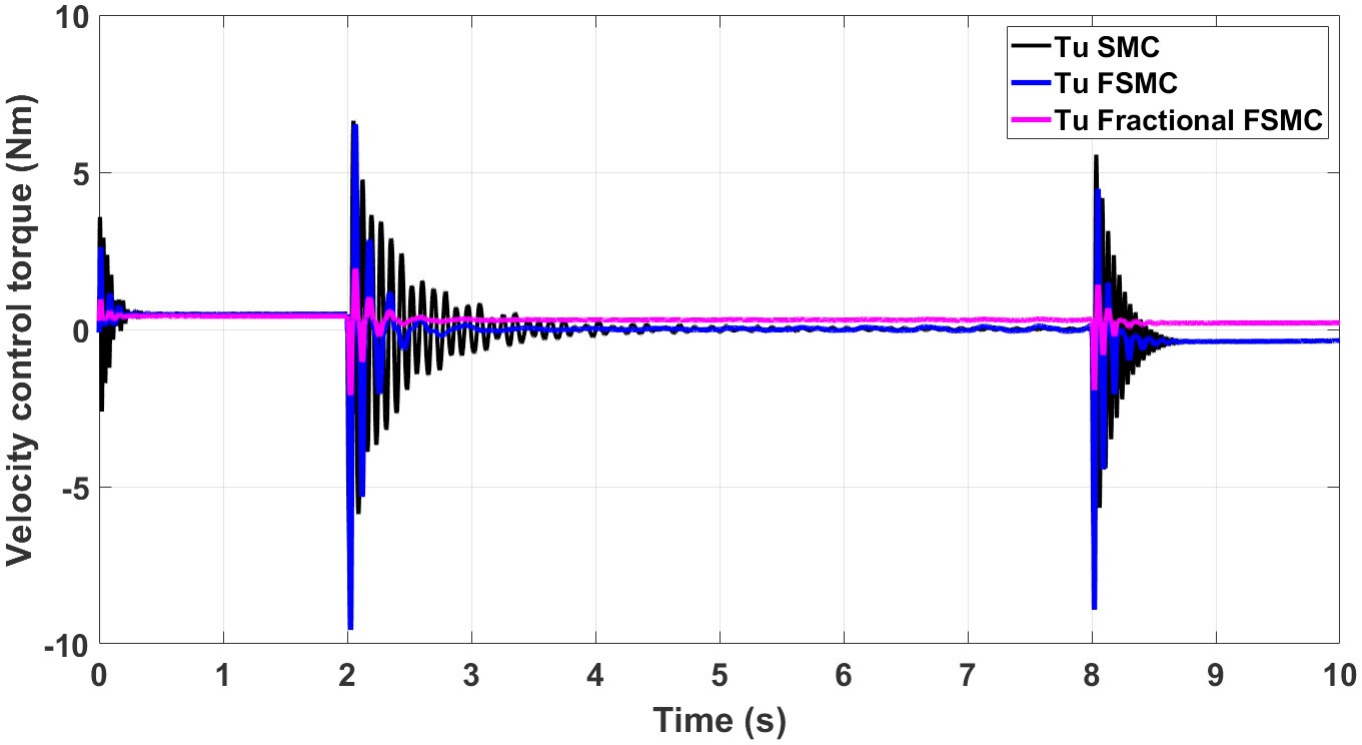
Velocity control torque response generated by SMC, FSMC and fractional FSMC based laws.

The generated control, yaw and velocity control torques are shown in Figs 16–18 respectively.

From the presented results in Figs 16–18, it is observed that the produced torques with SMC based law suffer from high frequency chattering in the event of acceleration and deceleration, while chattering is minimized in case of FSMC and fractional FSMC based controllers. Apart from chattering, the magnitudes of torques produced at various intervals of times are almost comparable for both SMC and FSMC based control schemes, while for fractional order FSMC, these magnitudes are smaller. Chattering is limited by 70% in FSMC and fractional FSMC schemes and the power usage and actuator life is increased due to reduced chattering. Hence, FSMC and fractional FSMC resolve the chattering issue and overcome the hindrance of constrained energy supply.

### Results and comparative study of SMC, FSMC and fractional FSMC with ramp yaw rate

**Fig 19:**
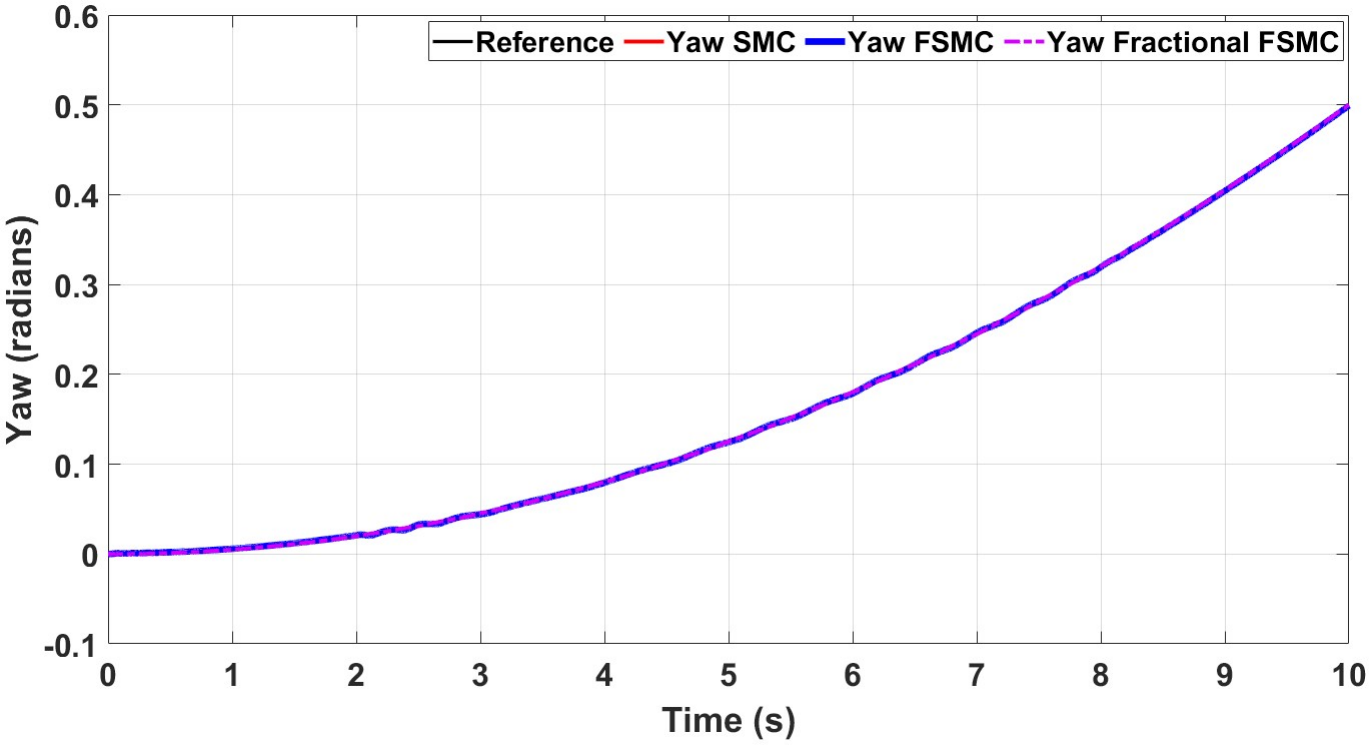
Yaw tracking comparison with ramp yaw rate as reference trajectory.

**Fig 20:**
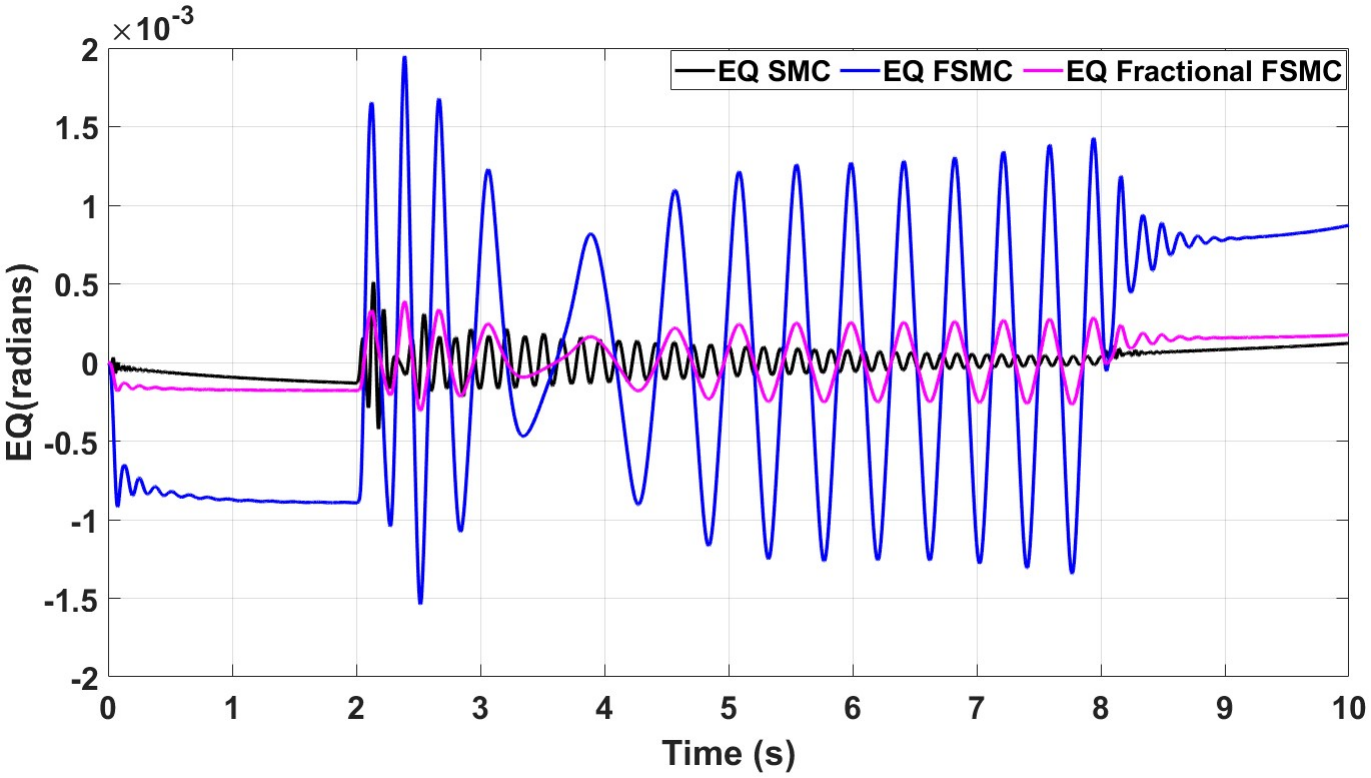
Comparison of yaw errors in case of SMC, FSMC and fractional FSMC under ramp yaw rate as reference.

**Fig 21:**
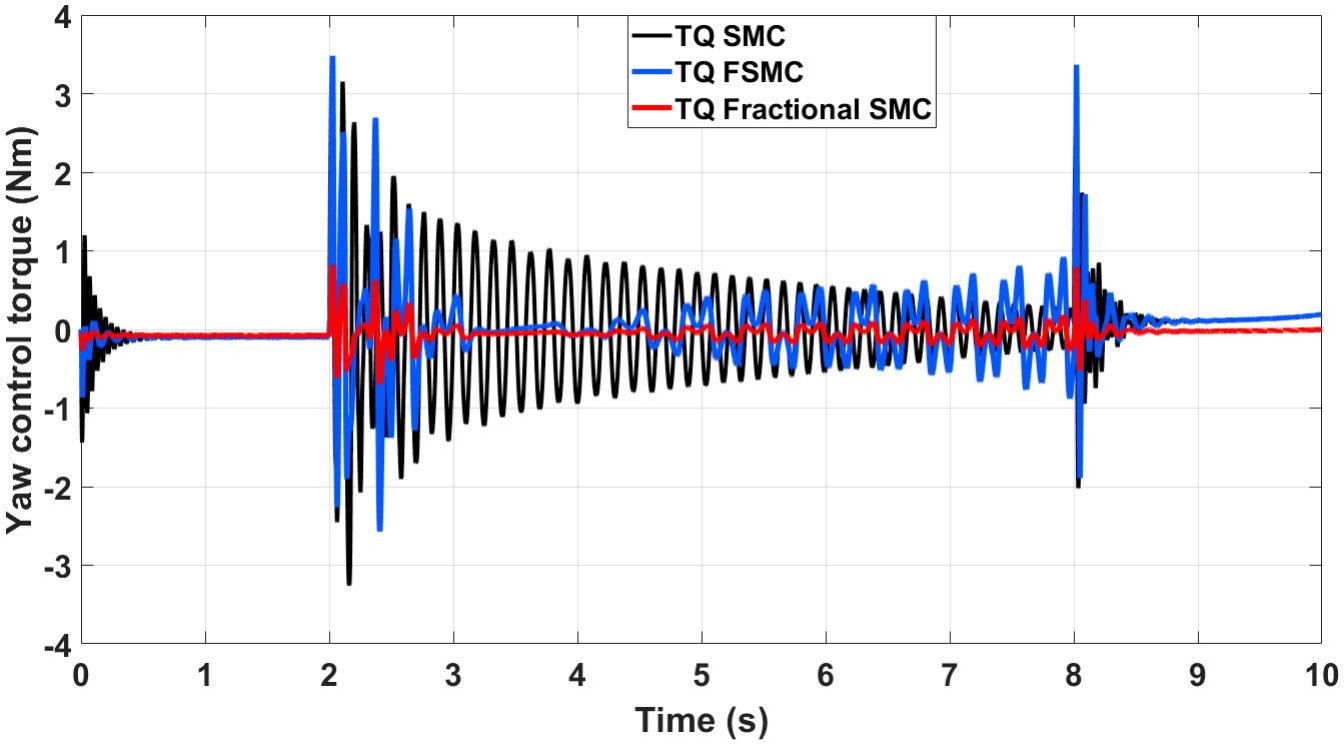
Comparison of yaw torque in case of SMC, FSMC and fractional FSMC under ramp yaw rate as reference.

The second test is conducted with ramp yaw rate. The reference yaw angle and system response under different control schemes are shown in Fig 19, while Figs 20 and 21 show the tracking errors and generated torque responses respectively. In Fig 19, the tracking comparison is done for Fractional FSMC, FSMC and SMC algorithms with ramp yaw rate as reference trajectory. Minimum errors are obtained for all the controllers as it tracks the reference trajectory but Fractional FSMC algorithm limits that error to almost zero. The maximum errors are obtained at time t = 2-3 seconds for all the controllers.

From the presented results in Fig 20, the magnitude of error is observed to be large with FSMC scheme. The lowest errors are ensured using SMC, while with fractional order SMC, the magnitude of errors is comparable to SMC. Secondly, the frequency of oscillations reduces in case of both FSMC and fractional order FSMC schemes. From Figs 14 and 15, and at time t = 2-4secs, the frequency of oscillations with SMC is twice as compared to the FSMC and fractional FSMC.

From the presented results in Fig 21, it is observed that the produced torques with SMC suffer from high frequency chattering in the event of acceleration and deceleration, while chattering is minimized in case of FSMC and fractional FSMC technique. Apart from chattering, the magnitudes of torques produced at various intervals of times are almost comparable for both SMC and FSMC schemes, while for fractional order FSMC, these magnitudes are smaller. Chattering is limited by 70% in both FSMC and fractional FSMC schemes and the power usage and actuator life is increased due to reduced chattering. Hence, FSMC and fractional FSMC resolve the chattering issue and overcome the hindrance of constrained energy supply.

**Fig 22:**
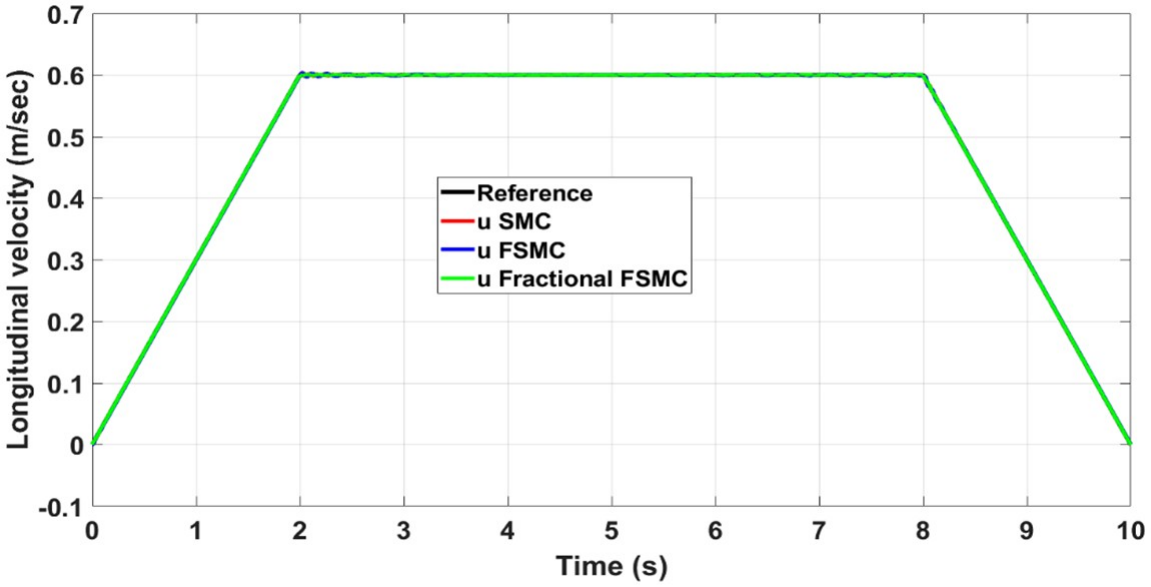
Longitudinal velocity tracking comparison under ramp yaw rate as reference.

**Fig 23:**
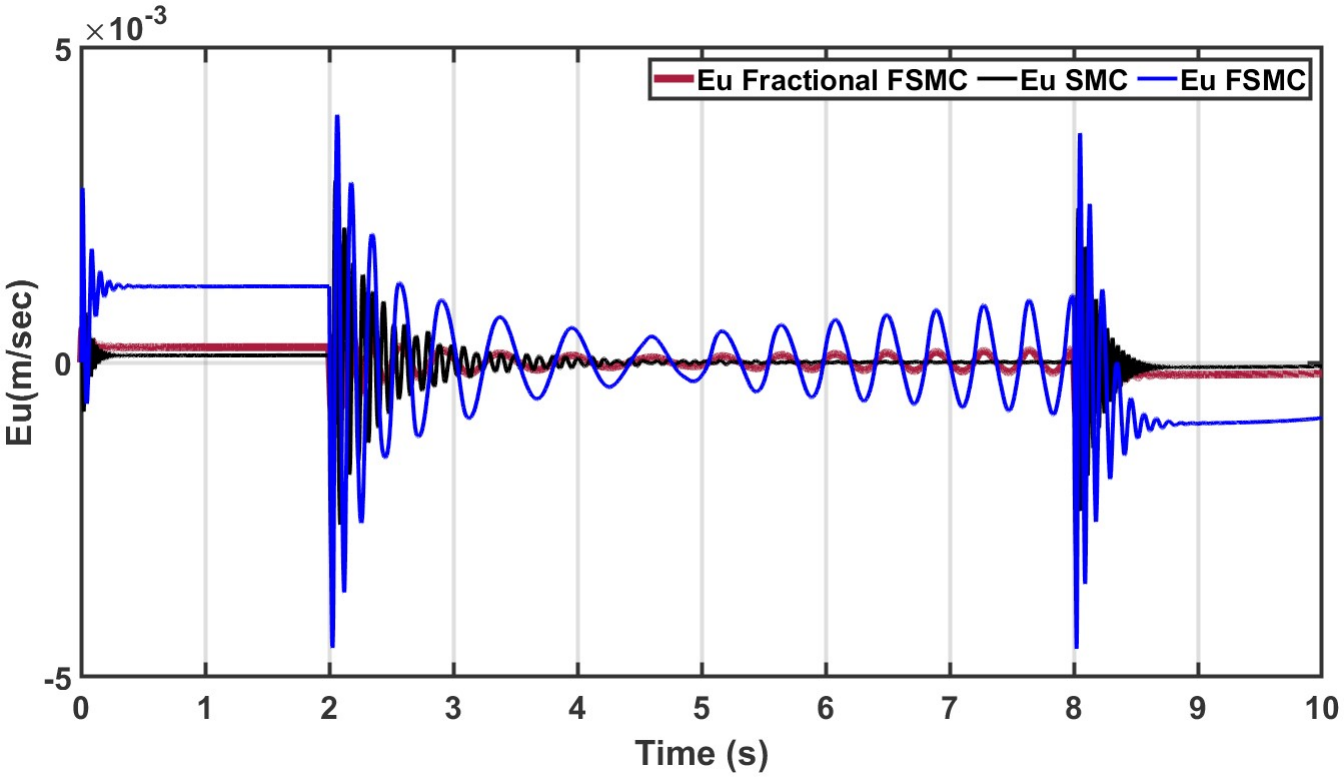
Longitudinal velocity error tracking comparison under ramp yaw rate as reference.

**Fig 24:**
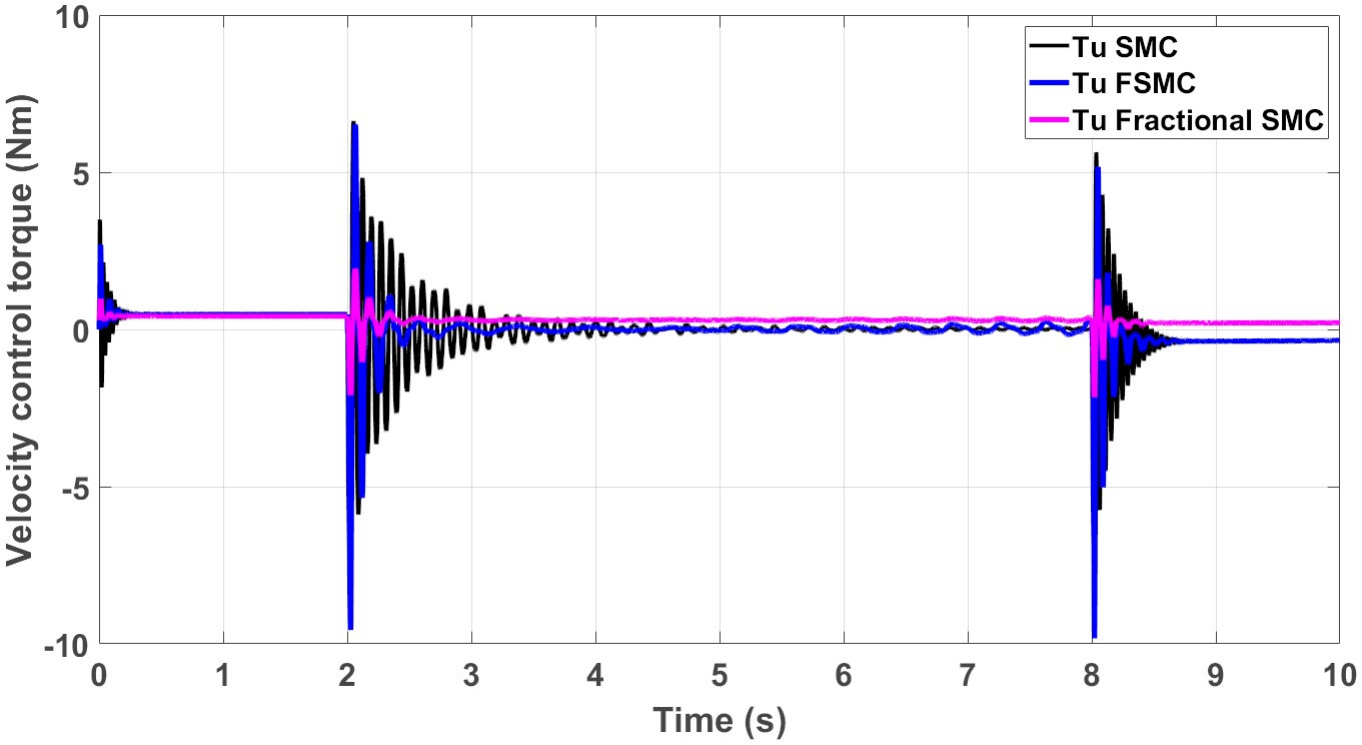
Longitudinal velocity torque comparison under ramp yaw rate as reference.

The longitudinal velocity tracking, the respective response errors and generated torques are shown in Figs 22–24 respectively. From the presented results, it is obvious that the fractional FSMC exhibits minimum chattering and also it ensured tracking errors that are comparable to SMC method.

The comparison of yaw and velocity errors with ramp yaw rate for SMC, FSMC and FFSMC algorithms is presented in Table 10. This table presents the yaw and velocity tracking errors of all the controllers at different times. It elaborates Figs 19 and 22 where data representation is difficult to present clearly.

**Table 10:** Yaw and velocity errors comparison with ramp yaw rate as reference.

Time (sec)	Yaw Error (Radians)	Velocity Error (m/sec)
	**SMC Algorithm**	
0	0.0001	1.25 × 10^−^3
2	0.0005	3.25 × 10^−^3
8	0.0002	3.1 × 10^−^3
	**FSMC Algorithm**	
0	0.0009	2.8 × 10^−^3
2	0.0016	4.5 × 10^−^3
8	0.0014	4.5 × 10^−^3
	**FFSMC Algorithm**	
0	0.0002	0.6 × 10^−^3
2	0.0003	0.9 × 10^−^3
8	0.00025	0.9 × 10^−^3

### Results and comparative study under varying friction coefficient for FSMC controller

**Fig 25:**
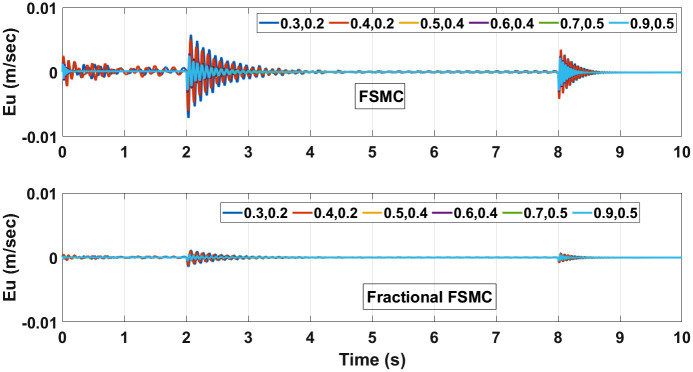
Velocity error at different coefficient of friction.

**Fig 26:**
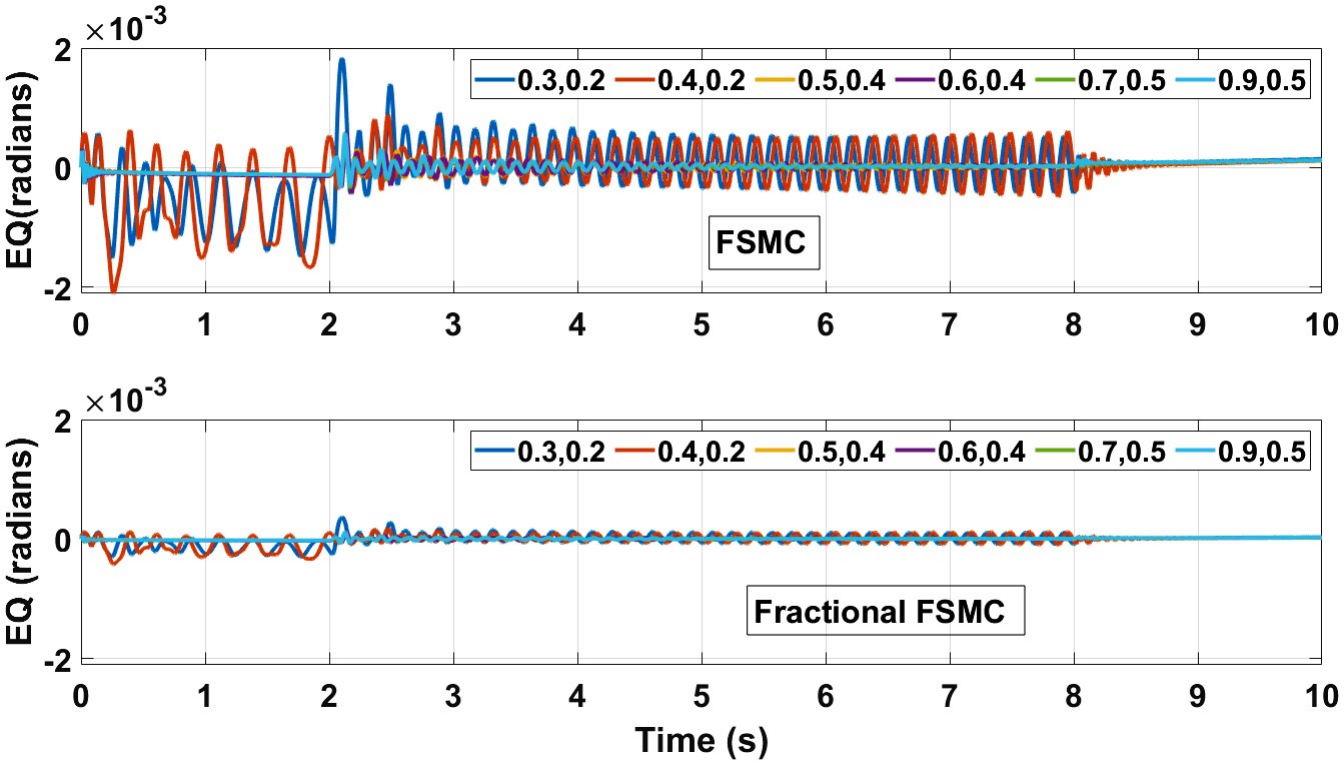
Yaw control error at different coefficient of friction.

The performance of FSMC and fractional FSMC controllers is evaluated by simulation analysis for varying coefficient of friction. Displaced CG and different friction coefficient values for different surfaces are considered and their effects are investigated. These surfaces include dry surfaces having high friction coefficient values and wet surfaces having low friction coefficient values. The first value in (*μ*_*x*_, *μ*_*y*_) represents the longitudinal slip and the second represents lateral slip for each surface. Figs 25 and 26 illustrate tracking error comparison of velocity and yaw angle errors. From the presented results, it is obvious that with different friction coefficients and displaced CG, the lowest errors are ensured using fractional FSMC, while in case of FSMC, the peak errors are large. The errors in fractional FSMC are very small due to its better performance. Table 11 enlists the recorded errors for FSMC from the error plots.

**Table 11:** Maximum yaw and velocity control errors at different friction coefficient for FSMC scheme.

u_*x*_, u_*y*_	Max yaw error (radians)	Max velocity error (m/sec)
0.3, 0.2	0.0018	0.0058
0.4, 0.2	8.8167e-04	0.0051
0.5, 0.4	5.2584e-04	0.0029
0.6, 0.4	5.9011e-04	0.0029
0.7, 0.5	4.9394e-04	0.0025
0.9, 0.5	5.8167e-04	0.0025

**Fig 27:**
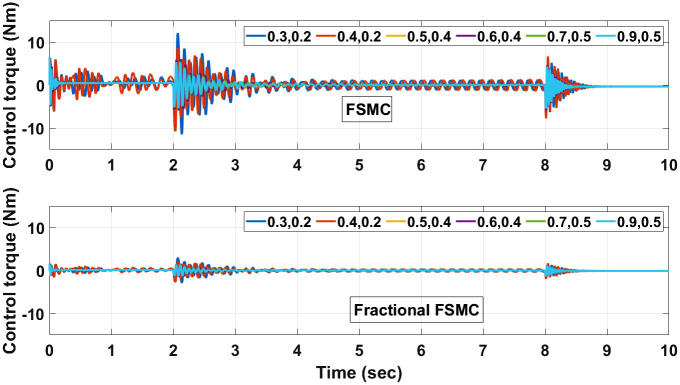
Control torque time response for SMC, FSMC and fractional FSMC.

**Fig 28:**
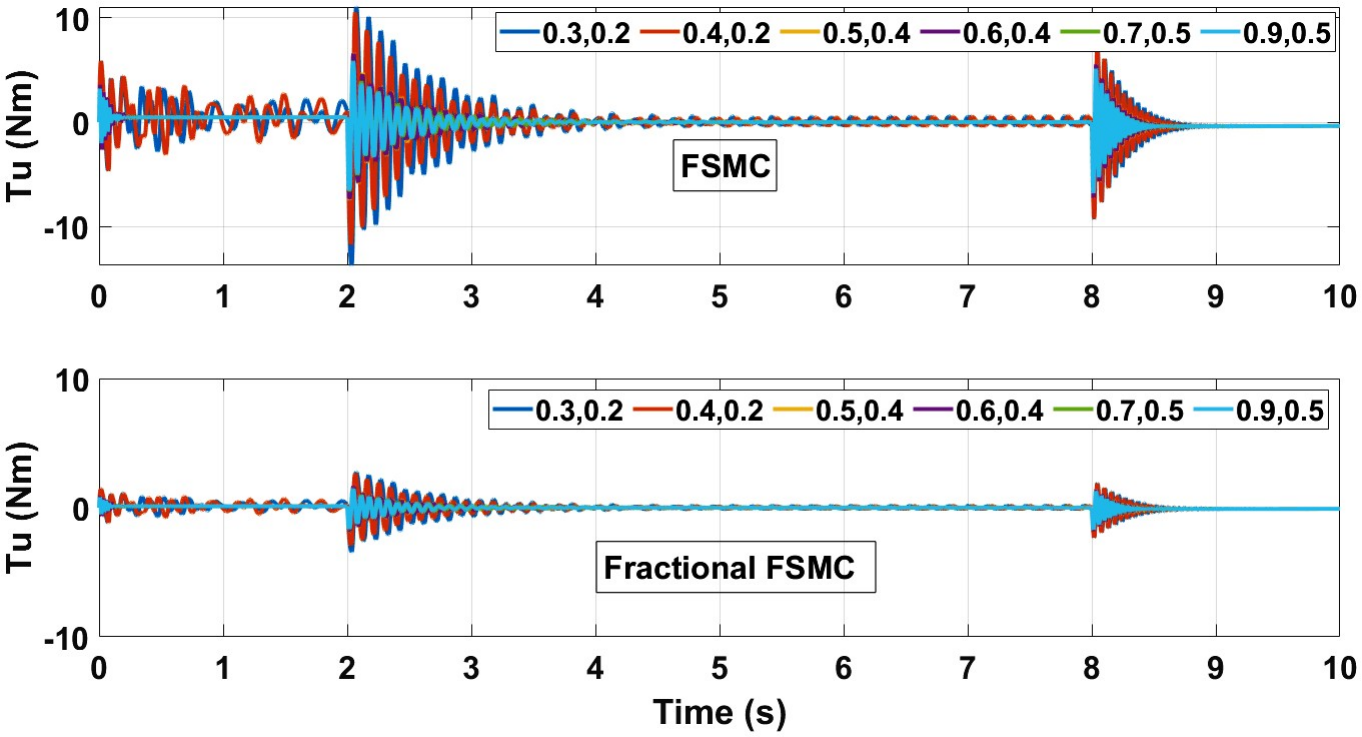
Velocity control torque time response for SMC, FSMC and fractional FSMC.

**Fig 29:**
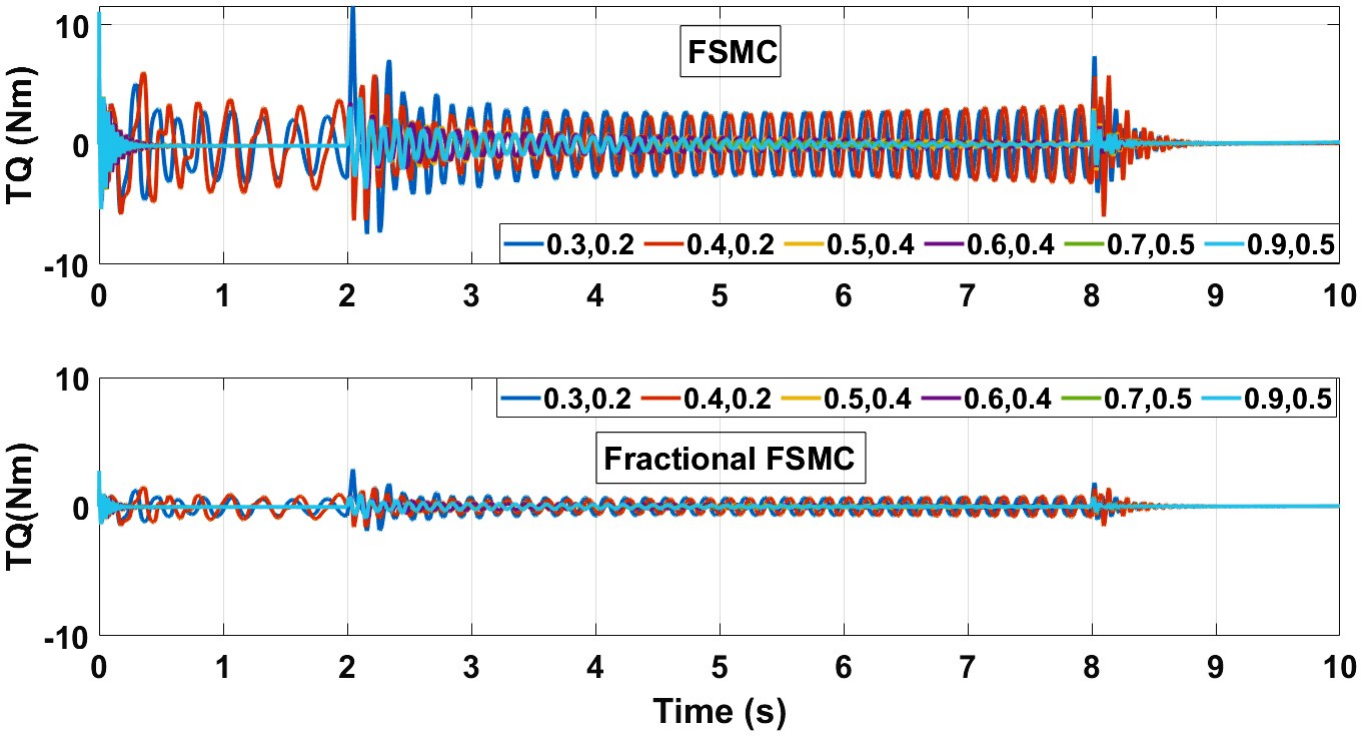
Yaw control torque time response for SMC, FSMC and fractional FSMC.

The control torques generated by FSMC and fractional FSMC schemes in response to the SSV curvatures for different friction coefficient are shown in Figs 27–29. From the presented results, it is obvious that under fractional order FSMC, the oscillations frequency reduces in the generated torques. This will reduce overall energy required to drive the motors.

## Conclusion

Integer and fractional order FSMC based schemes are proposed and implemented for the SSV vehicle. The proposed controllers are tested under variable CG and different ground friction coefficient. TM-Easy tire model is utilized, and robustness of the proposed controllers is evaluated with different road tire frictional coefficient and variable CG. From the presented results, it is concluded that the proposed fractional order FSMC controller reduces the power consumption and chattering with low friction coefficient and variable CG. Fractional order FSMC controller also reduces the yaw and velocity errors respectively as compared to FSMC and SMC controllers. Hence, the proposed Fractional order FSMC can be implemented on SSVs for robust performance and better power consumption.

## Abbreviations

*E*_*u*_: Velocity control Error
*E*_*θ*_: Yaw Control Torque
*F*_*x*_: Longitudinal Force
*F*_*y*_: Lateral Force
J: Moment of inertia of the SSV
*J*_*w*_: Moment of inertia of the wheel
M: Mass of the SSV
R: Radius of the tire
*S*_*x*_: Longitudinal Slip
*S*_*y*_: Lateral Slip
*V*_*x*_: Longitudinal Velocity
*V*_*y*_: Lateral Velocity
*τ*_*i*_: Control Input Torque
*τ*_*u*_: Velocity Control Torque
*τ*_*θ*_: Yaw Control Torque
